# Prevalence of Multiple Chronic Conditions Among Adults in the All of Us Research Program: Exploratory Analysis

**DOI:** 10.2196/69138

**Published:** 2025-05-12

**Authors:** Xintong Li, Caitlin Dreisbach, Carolina M Gustafson, Komal Patel Murali, Theresa A Koleck

**Affiliations:** 1 Goergen Institute for Data Science and Artificial Intelligence, University of Rochester, Rochester, NY, 14627, United States; 2 School of Nursing, University of Rochester, Rochester, NY, United States; 3 School of Nursing, University of Pittsburgh, Pittsburgh, PA, United States; 4 Rory Meyers College of Nursing, New York University, New York, NY, United States

**Keywords:** electronic health records, multiple chronic conditions, prevalence, secondary data analysis, United States, *All of Us*

## Abstract

**Background:**

The growing prevalence of multiple chronic conditions (MCC) has significant impacts on health care systems and quality of life. Understanding the prevalence of MCC throughout adulthood offers valuable insights into the evolving burden of chronic diseases and provides strategies for more effective health care outcomes.

**Objective:**

This study estimated the prevalence and combinations of MCC among adult participants enrolled in the *All of Us (AoU)* Research Program, especially studying the variations across age categories.

**Methods:**

We conducted an exploratory analysis using electronic health record (EHR) data from adult participants (N=242,828) in the version 7 Controlled Tier *AoU* Research Program data release. Data analysis was conducted using Python in a Jupyter notebook environment within the *AoU* Researcher Workbench. Descriptive statistics included condition frequencies, the number of chronic conditions per participant, and prevalence according to age categories. The presence of a chronic condition was determined by documentation of one or more *ICD-10* (*International Statistical Classification of Diseases, Tenth Revision*) codes for the respective condition. Age categories were established and aligned with diagnosis dates and stages of adulthood (early adulthood: 18-39 years; middle adulthood: 40-49 years; late middle adulthood: 50-64 years; late adulthood: 65-74 years; advanced old age: 75-89 years).

**Results:**

Our findings demonstrated that approximately 76% (n=183,753) of *AoU* participants were diagnosed with MCC, with over 40% (n=98,885) having 6 or more conditions and prevalence increasing with age (from 33.78% in early adulthood to 68.04% in advanced old age). The most frequently occurring MCC combinations varied by age category. Participants aged 18-39 years primarily presented mental health–related MCC combinations (eg, anxiety and depressive disorders; n=845), whereas those aged 40-64 years frequently had combinations of physical conditions such as fibromyalgia, chronic pain, fatigue, and arthritis (204 in middle adulthood and 457 in late middle adulthood). In late adulthood and advanced old age, hyperlipidemia and hypertension were the most commonly occurring MCC combinations (n=200 and n=59, respectively).

**Conclusions:**

We report notable prevalence of MCC throughout adulthood and variability in MCC combinations by age category in *AoU* participants. The significant prevalence of MCC underscores a considerable public health challenge, revealed by distinct condition combinations that shift across different life stages. Early adulthood is characterized predominantly by mental health conditions, transitioning to cardiometabolic and physical health conditions in middle, late, and advanced ages. These findings highlight the need for targeted, innovative care modalities and population health initiatives to address the burden of MCC throughout adulthood.

## Introduction

Multimorbidity is an increasingly prevalent global health challenge characterized by diverse, co-occurring chronic diseases, differing across life stages, demographics, and socioeconomic factors, necessitating shifts in health care practice and outcomes [1]. Multiple chronic conditions (MCC) is defined as the presence of two or more chronic conditions such as Alzheimer disease, arthritis, asthma, cancer, chronic kidney disease, diabetes, heart failure, and hypertension [2]. Approximately 80% of adults aged 65 years and older in the United States are living with MCC [3]. Older adults diagnosed with MCC often face significant care management challenges, including unplanned hospital readmissions and unmet care needs, which indicates the urgent need for enhanced support and tailored interventions for this vulnerable population [4]. Being diagnosed with MCC, however, is not limited to older adults. One study estimated that 22% of young adults, aged 18-34 years, are diagnosed with MCC [5]. Moreover, research suggests that more contemporary generations of adults have a greater MCC burden and are diagnosed with MCC at earlier ages than previous generations [6]. Estimation of the prevalence of MCC throughout all stages of adulthood is a critical reflection of the MCC burden; it is important to examine the prevalence of MCC broadly using regularly updated data sources to inform targeted prevention and management strategies and resource prioritization. Thus, the purpose of this study is to estimate the prevalence of MCC for adult participants in the National Institutes of Health’s *All of Us* (*AoU*) Research Program dataset—a curated disease and population-agnostic, longitudinal biomedical dataset of diverse individuals living in the United States—to gain a deeper understanding of MCC throughout adulthood (ie, early adulthood, middle adulthood, late middle adulthood, late adulthood, and advanced old age).

## Methods

### Participants

We conducted an exploratory cross-sectional analysis using the *AoU* Controlled Tier version 7 data release in the secure, cloud-based *AoU* Researcher Workbench platform (date of first access for research purposes: September 1, 2023; date of last access: June 16, 2024). Registered researchers can access the data through the *AoU* Research Hub [7]. This study was exempt from ethical approval for human subjects research as only deidentified data were analyzed.

Using the Cohort Builder tool, we identified all adult participants 18 years of age or older who consented to share electronic health record (EHR) data and had at least one *ICD-10* (*International Statistical Classification of Diseases, Tenth Revision*) code documented for any healthcare purpose (ie, not limited to a chronic condition-related code) to ensure the availability of EHR data (n=242,828). For all participants meeting these eligibility criteria, we extracted relevant *ICD-10* codes and the corresponding date of documentation for 58 chronic conditions (Multimedia Appendix 1) cataloged in the Centers for Medicare & Medicaid Services (CMS) Chronic Conditions Data Warehouse [8]. CMS supports a Chronic Conditions Data Warehouse that contains computable phenotypes for “common chronic conditions” plus “other chronic or potentially disabling conditions,” which include additional chronic health, mental health, and substance abuse conditions [9]. We operationalized the presence of a chronic condition as documentation of one or more *ICD-10* codes for the respective condition (Multimedia Appendix 1). Due to reliance on *ICD-10* codes for both cohort identification and as the source of condition information, we limited participant visits to on or after October 1, 2015, that is, initiation of *ICD-10* implementation. Thus, data included in this analysis are from October 1, 2015, to July 1, 2022, that is, latest date available in the version 7 release.

### Data Analysis

We used the Python programming language in Jupyter Notebook to chronologically order the chronic condition *ICD-10* codes by date of documentation for each participant. By distinguishing the onset date of the second unique chronic condition, we were able to identify participants with MCC. We calculated the number of participants with 0, 1, 2, 3, 4, 5, and 6+ chronic conditions in the overall cohort and stratified by age category, that is, early adulthood (18-39 years), middle adulthood (40-49 years), late middle adulthood (50-64 years), late adulthood (65-74 years), and advanced old age (75-89 years) [10,11]. Age categorization supports methodological requirements of analyses, promotes interpretability of results for clinicians and policy makers, and helps capture changes in physical health and social roles throughout adulthood. We used the age at first documentation of the second chronic condition for categorization. We used chi-square tests to assess differences in demographic variables between participants with 0 or 1 chronic condition and participants with MCC. This approach tested for differences in the frequencies of race, ethnicity, gender, marital status, education, health insurance status, and employment status. We used *P*<.05 to indicate statistical significance. Finally, we calculated the frequency of unique MCC combinations by the number of chronic conditions (ie, 2, 3, 4, 5, and 6+) and age category.

### Ethical Considerations

This study analyzed deidentified data from participants enrolled in the *AoU* Research Program. Participants in the *AoU* Research Program were recruited either in-person at *AoU* enrollment sites or on the *AoU* web-based portal [12]. Participants gave informed consent when they joined the program, agreeing that their anonymized data could be used broadly for biomedical research [12]. Enrollment and data collection of participants in the *AoU* program is approved by the *AoU* institutional review board (IRB) [13].

We analyzed the EHR data in the secure, cloud-based *AoU* Researcher Workbench. All data used in this analysis were deidentified without personal identification including shifted dates for categorization of age. Researchers included in this analysis were registered within the *AoU* Research Program and the Researcher Workbench, completed the required training for data access, and signed the Data User Code of Conduct. All analyses were performed following *AoU* policies and guidelines to ensure confidentiality and responsible use of data [14]. Analyses for this study did not require subsequent IRB approval because the study does not constitute research involving human subjects according to the *AoU* Research Program IRB [12]. The data used in this analysis do not include the elements necessary to identify individuals so the study is not subject to the Common Rule protections 45 CFR part 46 [15].

## Results

### Participants Statistics

**Figure 1.**
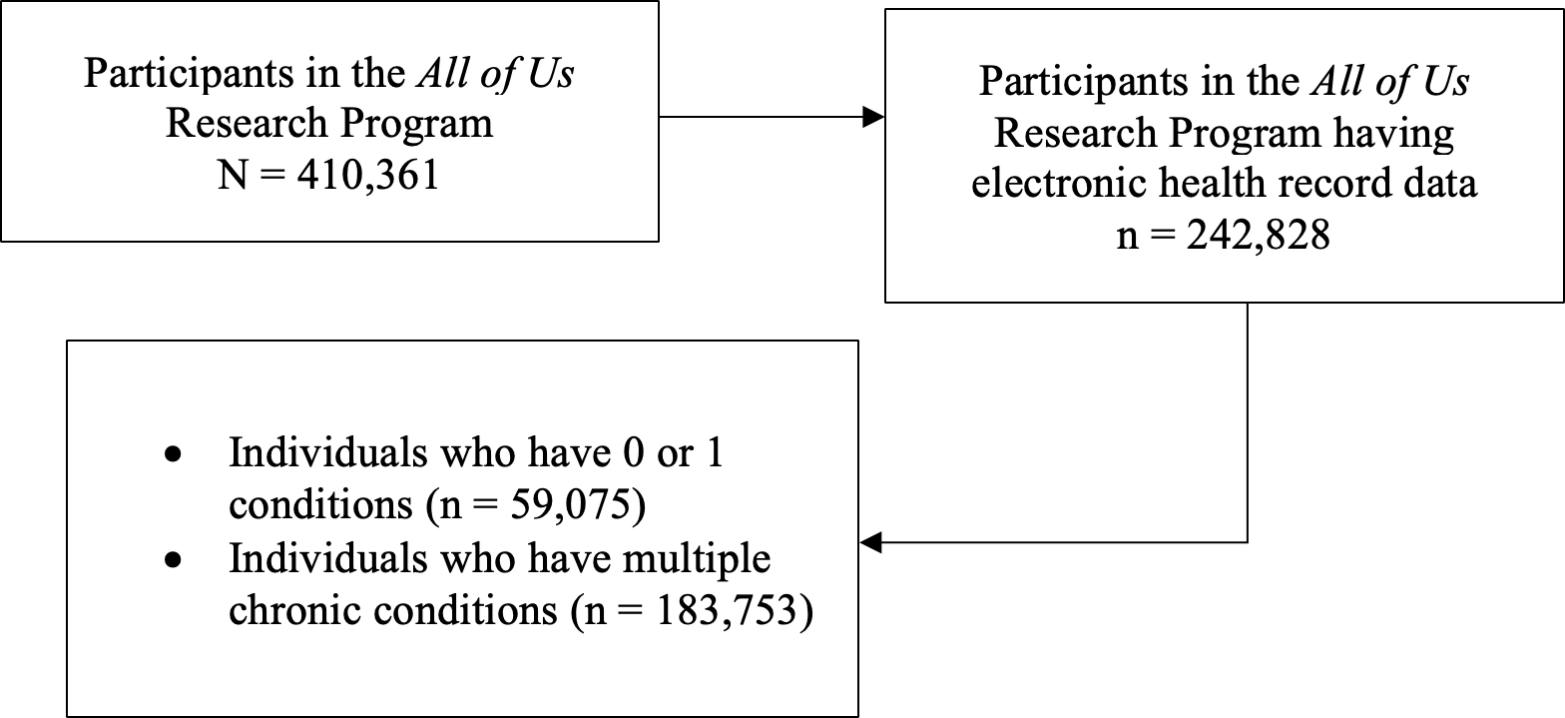
Flow diagram.

Approximately 76% (n=183,753) of participants were diagnosed with MCC (Figure 1), with over 40% of participants (n=98,885) diagnosed with 6 or more conditions (Table 1). Of the remaining participants, 11% (n=27,122) were diagnosed with a single chronic condition, and 13% (n=31,953) did not have a chronic condition documented. Overall, participants (Table 2) were on average 49.8 (SD 16.7) years of age and primarily women (n=147,991, 60.9%), White (n=137,039, 54.6%), not Hispanic or Latino (n=187,586, 77.3%), married or partnered (n=121,282, 49.9%), highly educated (n=166,815, 68.7% with some college or higher), insured (n=223,842, 92.2%), and not currently employed (n=111,229, 47.7%). Compared to participants diagnosed with 0 or 1 conditions, those diagnosed with MCC were older (age: mean 52.8 years vs 40.4 years). In addition, a higher percentage of participants with MCC were men (n=68,776, 37.4% vs n=19,956, 33.8%), White (n=107,725, 58.6% vs n=29,314, 49.6%), not Hispanic or Latino (n=145,393, 79.1% vs n=42,193, 71.4%), insured (n=170,985, 93.1% vs n=52,857, 89.5%), ever married (n=139,118, 75.7% vs n=39,264, 66.5%), and not currently employed (n=96,339, 52.4% vs n=19,484, 33.0%), with a much greater frequency of retired participants (n=48,565, 26.4% vs n=6025, 10.2%), compared to participants diagnosed with 0 or 1 conditions. The percentage of participants within an age category diagnosed with 6 or more chronic conditions increased with age (Table 1), from 33.78% in early adulthood to 68.04% in advanced old age.

**Table 1.** Prevalence of multiple chronic conditions by age group among adults enrolled in the *All of Us* Research Program.

Conditions		Participants, n (%)
**Overall (n=183,753)**		
	2	24,039 (9.90)
	3	21,910 (9.02)
	4	20,075 (8.27)
	5	18,844 (7.76)
	6+	98,885 (40.72)
**Early adulthood (18-39 years; n=40,237)**		
	2	9327 (23.18)
	3	7235 (17.98)
	4	5648 (14.04)
	5	4435 (11.02)
	6+	13,592 (33.78)
**Middle adulthood (40-49 years; n=28,906)**		
	2	4139 (14.32)
	3	3689 (12.76)
	4	3244 (11.22)
	5	3052 (10.56)
	6+	14,782 (51.14)
**Late middle adulthood (50-64 years; n=66,705)**		
	2	6786 (10.17)
	3	6945 (10.41)
	4	6895 (10.34)
	5	6715 (10.07)
	6+	39,364 (59.01)
**Late adulthood (65-74 years; n=35,549)**		
	2	2778 (7.81)
	3	3019 (8.49)
	4	3227 (9.08)
	5	3462 (9.74)
	6+	23,063 (64.88)
**Advanced old age (75-89 years; n=11,289)**		
	2	811 (7.18)
	3	844 (7.48)
	4	919 (8.14)
	5	1034 (9.16)
	6+	7681 (68.04)

**Table 2.** Characteristics of participants in the *All of Us* Research Program with the presence of multiple chronic conditions (2 or more chronic conditions; N=242,828).

		Overall (N=242,828)	0 or 1 condition (n=59,075)	Multiple chronic conditions (n=183,753)	*P* value^a^
Age^b^ (years), mean (SD)		49.8 (16.7)	40.4 (16.5)	52.8 (15.7)	<.001
**Race, n (%)**					<.001
	Asian	6514 (2.7)	2816 (4.8)	3698 (2.0)	
	Black	43,431 (17.9)	9991 (16.9)	33,440 (18.2)	
	Middle Eastern or North African	1413 (0.6)	462 (0.8)	951 (0.5)	
	Native Hawaiian or Pacific Islander	303 (0.1)	78 (0.1)	225 (0.1)	
	White	137,039 (56.4)	29,314 (49.6)	107,725 (58.6)	
	Multiracial	4233 (1.7)	1291 (2.2)	2942 (1.6)	
	Unknown	49,895 (20.5)	15,123 (25.6)	34,772 (18.9)	
**Ethnicity, n (%)**					<.001
	Not Hispanic or Latino	187,586 (77.3)	42,193 (71.4)	145,393 (79.1)	
	Hispanic or Latino	46,011 (18.9)	14,978 (25.4)	31,033 (16.9)	
	Unknown or not reported	9231 (3.8)	1904 (3.2)	7327 (4.0)	
**Gender, n (%)**					<.001
	Man	88,732 (36.5)	19,956 (33.8)	68,776 (37.4)	
	Woman	147,991 (60.9)	37,727 (63.9)	110,264 (60.0)	
	Nonbinary, transgender, or other gender	1622 (0.7)	479 (0.8)	1143 (0.6)	
	Unknown	4483 (1.8)	913 (1.5)	3570 (1.9)	
**Health insurance status, n (%)**					<.001
	Insured	223,842 (92.2)	52,857 (89.5)	170,985 (93.1)	
	Uninsured	11,268 (4.6)	4074 (6.9)	7194 (3.9)	
	Unknown	7718 (3.2)	2144 (3.6)	5574 (3.0)	
**Employment status, n (%)**					<.001
	Employed	99,435 (40.9)	31,910 (54.0)	67,525 (36.7)	
	Unemployed	20,477 (8.4)	5,634 (9.5)	14,843 (8.1)	
	Unable to work	27,250 (11.2)	2899 (4.9)	24,351 (13.3)	
	Retired	54,590 (22.5)	6025 (10.2)	48,565 (26.4)	
	Homemaker	8917 (3.7)	2704 (4.6)	6213 (3.4)	
	Student	4594 (1.9)	2515 (4.3)	2079 (1.1)	
	Multiple	18,653 (7.7)	4977 (8.4)	13,676 (7.4)	
	Unknown	8912 (3.7)	2411 (4.1)	6501 (3.5)	
**Annual income (US $), n (%)**					<.001
	Less than 10,000	31,274 (12.9)	7510 (12.7)	23,764 (12.9)	
	10,000 to 25,000	28,491 (11.7)	5702 (9.7)	22,789 (12.4)	
	25,000 to 35,000	17,271 (7.1)	4234 (7.2)	13,037 (7.1)	
	35,000 to 50,000	19,232 (7.9)	4769 (8.1)	14,463 (7.9)	
	50,000 to 75,000	25,428 (10.5)	6066 (10.3)	19,362 (10.5)	
	75,000 to 100,000	19,563 (8.1)	4752 (8.0)	14,811 (8.1)	
	100,000 to 150,000	23,765 (9.8)	6221 (10.5)	17,544 (9.5)	
	150,000 to 200,000	10,873 (4.5)	3020 (5.1)	7853 (4.3)	
	Greater than 200,000	15,186 (6.3)	4331 (7.3)	10,855 (5.9)	
	Unknown	51,745 (21.3)	12,470 (21.1)	39,275 (21.4)	
**Highest level of education, n (%)**					<.001
	Never attended	367 (0.2)	58 (0.1)	309 (0.2)	
	Up to high school	7710 (3.2)	1671 (2.8)	6039 (3.3)	
	Some high school	14,131 (5.8)	3351 (5.7)	10,780 (5.9)	
	High school graduate/GED	46,474 (19.1)	11,122 (18.8)	35,352 (19.2)	
	Some college	62,658 (25.8)	13,233 (22.4)	49,425 (26.9)	
	College graduate	53,993 (22.2)	15,107 (25.6)	38,886 (21.2)	
	Advanced degree	50,164 (20.7)	12,847 (21.7)	37,317 (20.3)	
	Unknown	7331 (3.0)	1686 (2.9)	5645 (3.1)	
**Marital status, n (%)**					<.001
	Married or living with partner	121,282 (49.9)	30,287 (51.3)	90,995 (49.5)	
	Divorced or separated	42,814 (17.6)	7351 (12.4)	35,463 (19.3)	
	Widowed	14,286 (5.9)	1626 (2.8)	12,660 (6.9)	
	Never married	56,246 (23.2)	17,710 (30.0)	38,536 (21.0)	
	Unknown	8200 (3.4)	2101 (3.6)	6099 (3.3)	

a *P* values calculated using chi-square tests indicate there is a significant difference in frequencies between participants with 0 or 1 chronic condition and those with multiple chronic conditions (2 or more conditions).

b Age (years) of participants on October 1, 2015, that is, the initiation of *ICD-10* (*International Statistical Classification of Diseases, Tenth Revision*) code implementation, for comparison between groups. Please note that age at first documentation of the second chronic condition, not age on October 1, 2015, was used for age categorization for participants diagnosed with multiple chronic conditions.

### Conditions Across Age Distributions

The top 5 most frequently diagnosed MCC combinations in each age category are detailed in Multimedia Appendix 1. The most frequently occurring combination in each age categorization was as follows: early adulthood: anxiety disorders and depression, bipolar disorder, or other depressive mood disorders (n=845); middle and late middle adulthood: fibromyalgia, chronic pain and fatigue, and rheumatoid arthritis or osteoarthritis (n=204 and n=457, respectively); and late adulthood and advanced old age: hyperlipidemia and hypertension (n=200 and n=59, respectively).

## Discussion

### Principal Findings

In this study, we estimated MCC prevalence in adults living in the United States from early adulthood to advanced old age using *ICD-10* codes from participants enrolled in the *AoU* program. We found that approximately 76% of participants have MCC, with higher prevalence as age increases. The most frequent MCC combinations varied notably by age category. Mental health conditions (ie, anxiety disorders and depression, bipolar disorder, or other depressive mood disorders) were most common in early adulthood, whereas combinations of physical health conditions (eg, fibromyalgia, chronic pain, fatigue, arthritis, hyperlipidemia, and hypertension) predominated in middle and older adulthood. These findings emphasize the value of the *AoU* program in identifying the MCC burden throughout adulthood. We believe the percentage of adults with MCC in this study is higher than that reported previously due to our robust inclusion of 58 chronic conditions, selected based on the CMS Chronic Conditions Data Warehouse.

### Comparison With Prior Work

Our results highlight distinct patterns in MCC combinations that vary by age category. Given the notable prevalence of MCC across adulthood, there is a continued need for innovative care modalities and public health initiatives to address the multifaceted nature of MCC [16]. MCC are associated with clinical complexity characterized by increased symptom burden, high health care usage and cost, and extensive caregiver strain [17]. The Agency for Healthcare Research and Quality states that care for people with MCC requires alignment among available resources, policies, and the workforce. For every dollar of health care spending in the United States, US $0.71 goes to treating individuals with MCC [3]. Subgroups at greater risk for MCC can benefit from early detection and prevention strategies, which may reduce hospital admissions and associated health care costs [3]. Ongoing research efforts that seek to both reduce the burden of MCC on the health care system and the development of person- and family-centered care strategies across the health care continuum are needed [17]. Public health leaders should coordinate efforts with clinicians, administrators, and communities to support changes in care management and outcomes across the lifespan.

We found that the most frequent MCC combinations vary by age category. Notably, a combination of mental, rather than physical, health conditions (ie, anxiety disorders and depression, bipolar disorder, or other depressive mood disorders) was the most common MCC combination in early adulthood. Following early adulthood, combinations of physical health conditions were the most common (eg, hyperlipidemia and hypertension in late adulthood and advanced old age). For precision health, characterizing MCC profiles and the most frequently co-occurring conditions in different age categories can inform targeted prevention and treatment approaches and allow clinicians to better anticipate and manage comorbidities in their patients [18].

### Limitations

We acknowledge, however, that this initial analysis is exploratory and descriptive in nature. The characteristics of adult participants who are enrolled in the *AoU* program and consented to share EHR data—ie, women, White individuals, non-Hispanic/Latino individuals, married/partnered individuals, highly educated individuals, insured individuals, and those not currently employed—must also be considered when interpreting the reported estimates and generalizing results to the broader US, and worldwide, population. Another limitation of our analysis is the reliance on *ICD-10* codes for identifying chronic conditions, which could introduce inaccuracies. Computable phenotyping challenges, such as accurate identification and measurement of chronic conditions, are not unique to this analysis but rather an inherent aspect of analysis using EHR data. Computable phenotypes for chronic conditions could be enhanced to reduce the risk of misdiagnosis and refine prevalence estimates. One approach to support the validity of chronic condition phenotypes would be comparison of CMS-derived diagnoses to EHR computable phenotypes from other repositories, such as the Observational Health Data Sciences and Informatics (OHDSI [19]) Phenotype Library and Phenotype KnowledgeBase (PheKB [20]). OHDSI and PheKB phenotypes use value sets including and beyond codes (eg, laboratory values and medications). Another approach to enhance the validity of chronic condition diagnoses would be to compare the computable phenotype–derived diagnoses to self-reported diagnoses on *AoU* surveys. Without a true gold standard, triangulation of EHR and survey sources may be an ideal strategy to achieve diagnosis accuracy in future work.

### Conclusions

This brief report fills an important knowledge gap and provides a foundation for future studies on MCC correlates, predictors, and outcomes by communicating current MCC prevalence estimates, which capture a wider range of chronic conditions, across the adult lifespan. The high prevalence of MCC in adults from early adulthood to advanced old age underscores the need for resource prioritization, targeted prevention, and improved treatment and care plans in individuals with co-occurring conditions.

## Supplementary material

10.2196/69138 Multimedia Appendix 1 Additional tables.

## Abbreviations

*AoU*: *All of Us*
CMS: Centers for Medicare & Medicaid Services
EHR: electronic health record
*ICD-10*: *International Statistical Classification of Diseases, Tenth Revision*
IRB: institutional review board
MCC: multiple chronic conditions
OHDSI: Observational Health Data Sciences and Informatics
PheKB: Phenotype Library and Phenotype KnowledgeBase
