# Supplementary material for: Prevalence of Multiple Chronic Conditions Among Adults in the All of Us Research Program: Exploratory Analysis
Source: JMIR Form Res. 2025 May 12;9:e69138. doi: 10.2196/69138 (PMC12088611; doi:10.2196/69138)
Supplement: Multimedia Appendix 1 [file formative-v9-e69138-s001.docx]

| Multimedia Appendix 2 - Supplementary Tables **Supplementary Table 1. Chronic conditions included in the multiple chronic condition prevalence analysis (N=58)** | |
| --- | --- |
| Acute myocardial infarction | HIV/AIDS |
| ADHD, conduct disorder, and hyperkinetic syndrome | Hyperlipidemia |
| Alzheimer’s disease | Hypertension |
| Anemia^1^ | Hypothyroidism |
| Anxiety disorders^2^ | Intellectual disabilities and related conditions |
| Asthma | Ischemic heart disease |
| Atrial fibrillation and flutter | Learning disabilities and developmental delays^3^ |
| Autism spectrum disorders | Leukemias and lymphomas |
| Benign prostatic hyperplasia | Liver disease, cirrhosis and other liver conditions |
| Blindness and visual impairment | Migraine and chronic headache |
| Cancer, breast | Mobility impairments |
| Cancer, colorectal | Multiple sclerosis and transverse myelitis |
| Cancer, endometrial | Muscular dystrophy |
| Cancer, lung | Non-Alzheimer’s dementia |
| Cancer, prostate | Obesity |
| Cancer, urologic | Osteoporosis |
| Cataract | Parkinson’s disease and secondary parkinsonism |
| Cerebral palsy | Peripheral vascular disease |
| Chronic kidney disease | Personality disorders |
| Chronic obstructive pulmonary disease | Pneumonia |
| Cystic fibrosis and other metabolic developmental disorders | Pressure and chronic ulcers |
| Deafness and hearing impairment | Rheumatoid arthritis/osteoarthritis |
| Depression, bipolar, or other depressive mood disorders | Schizophrenia and other psychotic disorders |
| Diabetes | Spina bifida and other congenital anomalies of the nervous system |
| Epilepsy | Spinal cord injury |
| Fibromyalgia, chronic pain and fatigue | Stroke/transient ischemic attack |
| Glaucoma | Substance use disorders^4^ |
| Heart failure and non-ischemic heart disease | Traumatic brain injury and nonpsychotic mental disorders due to brain damage |
| Hip/pelvic fracture | Viral hepatitis |
| ADHD=Attention-deficit/hyperactivity disorder; HIV/AIDS=Human immunodeficiency virus/Acquired immunodeficiency syndrome.  International Classification of Disease-10 Revision (ICD-10) codes from the Centers for Medicare and Medicaid Services Chronic Condition Warehouse were used identify diagnosis of a chronic condition (5-7).  ^1^Includes ICD-10 codes for “anemia” and “sickle cell anemia”  ^2^Includes ICD-10 codes for “anxiety disorders” and “post-traumatic stress disorder”  ^3^Includes ICD-10 codes for “learning disabilities” and “other developmental delays”  ^4^Includes ICD-10 codes for “alcohol use disorders”; “drug use disorders”; opioid use disorder 1, 2, 3 and 4; and “tobacco use disorders”, excluding Z71.41, Z71.42, Z71.51, Z71.52, and Z71.6 | |

| **Supplementary Table 2.**  **International Classification of Disease-10 Revision (ICD-10) codes from the Centers for Medicare and Medicaid Services Chronic Condition Warehouse used identify diagnosis of each chronic condition** | |
| --- | --- |
| Acute myocardial infarction | I21.01 \| I21.02 \| I21.09 \| I21.11 \| I21.19 \| I21.21 \| I21.29 \| I21.3 \| I21.4 \| I21.9 \| I21.A1 \| I21.A9 \| I22.0 \| I22.1 \| I22.2 \| I22.8 \| I22.9 \| I23.0 \| I23.1 \| I23.2 \| I23.3 \| I23.4 \| I23.5 \| I23.6 \| I23.7 \| I23.8 |
| ADHD, conduct disorder, and hyperkinetic syndrome | F63.0 \| F63.1 \| F63.2 \| F63.3 \| F63.81 \| F63.89 \| F63.9 \| F90.0 \| F90.1 \| F90.2 \| F90.8 \| F90.9 \| F91.0 \| F91.1 \| F91.2 \| F91.3 \| F91.8 \| F91.9 |
| Alzheimer’s disease | G30.0 \| G30.1 \| G30.8 \| G30.9 |
| Anemia^1^ | C94.6 \| D46.0 \| D46.1 \| D46.20 \| D46.21 \| D46.22 \| D46.4 \| D46.9 \| D46.A \| D46.B \| D46.C \| D46.Z \| D47.4 \| D50.0 \| D50.1 \| D50.8 \| D50.9 \| D51.0 \| D51.1 \| D51.2 \| D51.3 \| D51.8 \| D51.9 \| D52.0 \| D52.1 \| D52.8 \| D52.9 \| D53.0 \| D53.1 \| D53.2 \| D53.8 \| D53.9 \| D55.0 \| D55.1 \| D55.2 \| D55.21 \| D55.29 \| D55.3 \| D55.8 \| D55.9 \| D56.0 \| D56.1 \| D56.2 \| D56.3 \| D56.4 \| D56.5 \| D56.8 \| D56.9 \| D57.00 \| D57.01 \| D57.02 \| D57.03 \| D57.09 \| D57.1 \| D57.20 \| D57.211 \| D57.212 \| D57.213 \| D57.218 \| D57.219 \| D57.3 \| D57.40 \| D57.411 \| D57.412 \| D57.413 \| D57.418 \| D57.419 \| D57.42 \| D57.431 \| D57.432 \| D57.433 \| D57.438 \| D57.439 \| D57.44 \| D57.451 \| D57.452 \| D57.453 \| D57.458 \| D57.459 \| D57.80 \| D57.811 \| D57.812 \| D57.813 \| D57.818 \| D57.819 \| D58.0 \| D58.1 \| D58.2 \| D58.8 \| D58.9 \| D59.0 \| D59.1 \| D59.10 \| D59.11 \| D59.12 \| D59.13 \| D59.19 \| D59.2 \| D59.3 \| D59.30 \| D59.31 \| D59.32 \| D59.39 \| D59.4 \| D59.5 \| D59.6 \| D59.8 \| D59.9 \| D60.0 \| D60.1 \| D60.8 \| D60.9 \| D61.01 \| D61.09 \| D61.1 \| D61.2 \| D61.3 \| D61.810 \| D61.811 \| D61.818 \| D61.82 \| D61.89 \| D61.9 \| D63.0 \| D63.1 \| D63.8 \| D64.0 \| D64.1 \| D64.2 \| D64.3 \| D64.4 \| D64.81 \| D64.89 \| D64.9 \| D75.81 |
| Anxiety disorders^2^ | F06.4 \| F40.00 \| F40.01 \| F40.02 \| F40.10 \| F40.11 \| F40.210 \| F40.218 \| F40.220 \| F40.228 \| F40.230 \| F40.231 \| F40.232 \| F40.233 \| F40.240 \| F40.241 \| F40.242 \| F40.243 \| F40.248 \| F40.290 \| F40.291 \| F40.298 \| F40.8 \| F40.9 \| F41.0 \| F41.1 \| F41.3 \| F41.8 \| F41.9 \| F42 \| F42.2 \| F42.3 \| F42.4 \| F42.8 \| F42.9 \| F43.0 \| F43.10 \| F43.11 \| F43.12 \| F44.9 \| F45.8 \| F48.8 \| F48.9 |
| Asthma | J45.20 \| J45.21 \| J45.22 \| J45.30 \| J45.31 \| J45.32 \| J45.40 \| J45.41 \| J45.42 \| J45.50 \| J45.51 \| J45.52 \| J45.901 \| J45.902 \| J45.909 \| J45.990 \| J45.991 \| J45.998 |
| Atrial fibrillation and flutter | I48.0 \| I48.1 \| I48.11 \| I48.19 \| I48.2 \| I48.20 \| I48.21 \| I48.3 \| I48.4 \| I48.91 |
| Autism spectrum disorders | F84.0 \| F84.3 \| F84.5 \| F84.8 \| F84.9 |
| Benign prostatic hyperplasia | N40.0 \| N40.1 \| N40.2 \| N40.3 |
| Blindness and visual impairment | H54.0 \| H54.0X33 \| H54.0X34 \| H54.0X35 \| H54.0X43 \| H54.0X44 \| H54.0X45 \| H54.0X53 \| H54.0X54 \| H54.0X55 \| H54.10 \| H54.11 \| H54.1131 \| H54.1132 \| H54.1141 \| H54.1142 \| H54.1151 \| H54.1152 \| H54.12 \| H54.1213 \| H54.1214 \| H54.1215 \| H54.1223 \| H54.1224 \| H54.1225 \| H54.2 \| H54.2X11 \| H54.2X12 \| H54.2X21 \| H54.2X22 \| H54.3 \| H54.8 |
| Cancer, breast | C50.011 \| C50.012 \| C50.019 \| C50.021 \| C50.022 \| C50.029 \| C50.111 \| C50.112 \| C50.119 \| C50.121 \| C50.122 \| C50.129 \| C50.211 \| C50.212 \| C50.219 \| C50.221 \| C50.222 \| C50.229 \| C50.311 \| C50.312 \| C50.319 \| C50.321 \| C50.322 \| C50.329 \| C50.411 \| C50.412 \| C50.419 \| C50.421 \| C50.422 \| C50.429 \| C50.511 \| C50.512 \| C50.519 \| C50.521 \| C50.522 \| C50.529 \| C50.611 \| C50.612 \| C50.619 \| C50.621 \| C50.622 \| C50.629 \| C50.811 \| C50.812 \| C50.819 \| C50.821 \| C50.822 \| C50.829 \| C50.911 \| C50.912 \| C50.919 \| C50.921 \| C50.922 \| C50.929 \| D05.00 \| D05.01 \| D05.02 \| D05.10 \| D05.11 \| D05.12 \| D05.80 \| D05.81 \| D05.82 \| D05.90 \| D05.91 \| D05.92 \| Z17.0 \| Z17.1 \| Z19.1 \| Z19.2 \| Z85.3 \| Z86.000 |
| Cancer, colorectal | C18.0 \| C18.1 \| C18.2 \| C18.3 \| C18.4 \| C18.5 \| C18.6 \| C18.7 \| C18.8 \| C18.9 \| C19 \| C20 \| C49.A4 \| C49.A5 \| D01.0 \| D01.1 \| D01.2 \| Z85.030 \| Z85.038 \| Z85.040 \| Z85.048 |
| Cancer, endometrial | C54.0 \| C54.1 \| C54.2 \| C54.3 \| C54.8 \| C54.9 \| D07.0 \| Z85.42 |
| Cancer, lung | C34.00 \| C34.01 \| C34.02 \| C34.10 \| C34.11 \| C34.12 \| C34.2 \| C34.30 \| C34.31 \| C34.32 \| C34.80 \| C34.81 \| C34.82 \| C34.90 \| C34.91 \| C34.92 \| D02.20 \| D02.21 \| D02.22 \| Z85.110 \| Z85.118 |
| Cancer, prostate | C61 \| D07.5 \| Z85.46 |
| Cancer, urologic | C64.1 \| C64.2 \| C64.9 \| C65.1 \| C65.2 \| C65.9 \| C66.1 \| C66.2 \| C66.9 \| C68.8 \| C68.9 \| D09.10 \| D09.19 \| Z85.520 \| Z85.528 \| Z85.53 \| Z85.54 \| Z85.59 |
| Cataract | H25.011 \| H25.012 \| H25.013 \| H25.019 \| H25.031 \| H25.032 \| H25.033 \| H25.039 \| H25.041 \| H25.042 \| H25.043 \| H25.049 \| H25.091 \| H25.092 \| H25.093 \| H25.099 \| H25.10 \| H25.11 \| H25.12 \| H25.13 \| H25.20 \| H25.21 \| H25.22 \| H25.23 \| H25.811 \| H25.812 \| H25.813 \| H25.819 \| H25.89 \| H25.9 \| H26.001 \| H26.002 \| H26.003 \| H26.009 \| H26.011 \| H26.012 \| H26.013 \| H26.019 \| H26.031 \| H26.032 \| H26.033 \| H26.039 \| H26.041 \| H26.042 \| H26.043 \| H26.049 \| H26.051 \| H26.052 \| H26.053 \| H26.059 \| H26.061 \| H26.062 \| H26.063 \| H26.069 \| H26.09 \| H26.101 \| H26.102 \| H26.103 \| H26.109 \| H26.111 \| H26.112 \| H26.113 \| H26.119 \| H26.121 \| H26.122 \| H26.123 \| H26.129 \| H26.131 \| H26.132 \| H26.133 \| H26.139 \| H26.20 \| H26.211 \| H26.212 \| H26.213 \| H26.219 \| H26.221 \| H26.222 \| H26.223 \| H26.229 \| H26.30 \| H26.31 \| H26.32 \| H26.33 \| H26.40 \| H26.411 \| H26.412 \| H26.413 \| H26.419 \| H26.491 \| H26.492 \| H26.493 \| H26.499 \| H26.8 \| H26.9 \| Q12.0 |
| Cerebral palsy | G80.0 \| G80.1 \| G80.2 \| G80.3 \| G80.4 \| G80.8 \| G80.9 |
| Chronic kidney disease | A18.11 \| A52.75 \| B52.0 \| I12.0 \| I12.9 \| I13.0 \| I13.10 \| I13.11 \| I13.2 \| M10.30 \| M10.311 \| M10.312 \| M10.319 \| M10.321 \| M10.322 \| M10.329 \| M10.331 \| M10.332 \| M10.339 \| M10.341 \| M10.342 \| M10.349 \| M10.351 \| M10.352 \| M10.359 \| M10.361 \| M10.362 \| M10.369 \| M10.371 \| M10.372 \| M10.379 \| M10.38 \| M10.39 \| M32.14 \| M32.15 \| M35.04 \| M35.0A \| N01.0 \| N01.1 \| N01.2 \| N01.3 \| N01.4 \| N01.5 \| N01.6 \| N01.7 \| N01.8 \| N01.9 \| N01.A \| N02.0 \| N02.1 \| N02.2 \| N02.3 \| N02.4 \| N02.5 \| N02.6 \| N02.7 \| N02.8 \| N02.9 \| N02.A \| N03.0 \| N03.1 \| N03.2 \| N03.3 \| N03.4 \| N03.5 \| N03.6 \| N03.7 \| N03.8 \| N03.9 \| N03.A \| N04.0 \| N04.1 \| N04.2 \| N04.3 \| N04.4 \| N04.5 \| N04.6 \| N04.7 \| N04.8 \| N04.9 \| N04.A \| N05.0 \| N05.1 \| N05.2 \| N05.3 \| N05.4 \| N05.5 \| N05.6 \| N05.7 \| N05.8 \| N05.9 \| N05.A \| N06.0 \| N06.1 \| N06.2 \| N06.3 \| N06.4 \| N06.5 \| N06.6 \| N06.7 \| N06.8 \| N06.9 \| N06.A \| N07.0 \| N07.1 \| N07.2 \| N07.3 \| N07.4 \| N07.5 \| N07.6 \| N07.7 \| N07.8 \| N07.9 \| N07.A \| N08 \| N14.0 \| N14.1 \| N14.11 \| N14.19 \| N14.2 \| N14.3 \| N14.4 \| N15.0 \| N15.8 \| N15.9 \| N16 \| N18.1 \| N18.2 \| N18.3 \| N18.30 \| N18.31 \| N18.32 \| N18.4 \| N18.5 \| N18.6 \| N18.9 \| N25.1 \| N25.89 \| N25.9 \| N26.1 \| N26.9 \| N99.0 \| Q61.02 \| Q61.11 \| Q61.19 \| Q61.2 \| Q61.3 \| Q61.4 \| Q61.5 \| Q61.8 |
| Chronic obstructive pulmonary disease | J40 \| J41.0 \| J41.1 \| J41.8 \| J42 \| J43.0 \| J43.1 \| J43.2 \| J43.9 \| J44.0 \| J44.1 \| J44.9 \| J47.0 \| J47.1 \| J47.9 \| J98.2 \| J98.3 |
| Cystic fibrosis and other metabolic developmental disorders | D81.810 \| D84.1 \| E25.0 \| E25.8 \| E25.9 \| E56.9 \| E70.0 \| E70.1 \| E70.20 \| E70.21 \| E70.29 \| E70.30 \| E70.310 \| E70.311 \| E70.318 \| E70.319 \| E70.320 \| E70.321 \| E70.328 \| E70.329 \| E70.330 \| E70.331 \| E70.338 \| E70.339 \| E70.39 \| E70.5 \| E70.8 \| E70.81 \| E70.89 \| E70.9 \| E71.0 \| E71.110 \| E71.111 \| E71.118 \| E71.19 \| E71.2 \| E71.310 \| E71.311 \| E71.312 \| E71.313 \| E71.314 \| E71.318 \| E71.32 \| E71.41 \| E72.10 \| E72.11 \| E72.12 \| E72.19 \| E72.20 \| E72.21 \| E72.22 \| E72.23 \| E72.29 \| E72.3 \| E72.4 \| E72.50 \| E72.51 \| E72.59 \| E72.8 \| E74.20 \| E74.21 \| E74.29 \| E74.810 \| E74.818 \| E74.819 \| E74.89 \| E84.0 \| E84.11 \| E84.19 \| E84.8 \| E84.9 |
| Deafness and hearing impairment | H90.3 \| H90.41 \| H90.42 \| H90.5 \| H90.6 \| H90.71 \| H90.72 \| H90.8 \| H90.A21 \| H90.A22 \| H90.A31 \| H90.A32 \| H91.01 \| H91.02 \| H91.03 \| H91.09 \| H91.3 \| H91.8X1 \| H91.8X2 \| H91.8X3 \| H91.8X9 \| H91.90 \| H91.91 \| H91.92 \| H91.93 |
| Depression, bipolar, or other depressive mood disorders | F06.31 \| F06.32 \| F31.0 \| F31.10 \| F31.11 \| F31.12 \| F31.13 \| F31.2 \| F31.30 \| F31.31 \| F31.32 \| F31.4 \| F31.5 \| F31.60 \| F31.61 \| F31.62 \| F31.63 \| F31.64 \| F31.71 \| F31.73 \| F31.75 \| F31.76 \| F31.77 \| F31.78 \| F31.81 \| F31.89 \| F31.9 \| F32.0 \| F32.1 \| F32.2 \| F32.4 \| F32.5 \| F32.8 \| F32.89 \| F32.9 \| F32.A \| F33 \| F33.0 \| F33.1 \| F33.2 \| F33.3 \| F33.4 \| F33.40 \| F33.41 \| F33.42 \| F33.8 \| F33.9 \| F34.1 \| F43.21 \| F43.23 |
| Diabetes | E08.00 \| E08.01 \| E08.10 \| E08.11 \| E08.21 \| E08.22 \| E08.29 \| E08.311 \| E08.319 \| E08.321 \| E08.3211 \| E08.3212 \| E08.3213 \| E08.3219 \| E08.329 \| E08.3291 \| E08.3292 \| E08.3293 \| E08.3299 \| E08.331 \| E08.3311 \| E08.3312 \| E08.3313 \| E08.3319 \| E08.339 \| E08.3391 \| E08.3392 \| E08.3393 \| E08.3399 \| E08.341 \| E08.3411 \| E08.3412 \| E08.3413 \| E08.3419 \| E08.349 \| E08.3491 \| E08.3492 \| E08.3493 \| E08.3499 \| E08.351 \| E08.3511 \| E08.3512 \| E08.3513 \| E08.3519 \| E08.3521 \| E08.3522 \| E08.3523 \| E08.3529 \| E08.3531 \| E08.3532 \| E08.3533 \| E08.3539 \| E08.3541 \| E08.3542 \| E08.3543 \| E08.3549 \| E08.3551 \| E08.3552 \| E08.3553 \| E08.3559 \| E08.359 \| E08.3591 \| E08.3592 \| E08.3593 \| E08.3599 \| E08.36 \| E08.37X1 \| E08.37X2 \| E08.37X3 \| E08.37X9 \| E08.39 \| E08.40 \| E08.41 \| E08.42 \| E08.43 \| E08.44 \| E08.49 \| E08.51 \| E08.52 \| E08.59 \| E08.610 \| E08.618 \| E08.620 \| E08.621 \| E08.622 \| E08.628 \| E08.630 \| E08.638 \| E08.641 \| E08.649 \| E08.65 \| E08.69 \| E08.8 \| E08.9 \| E09.00 \| E09.01 \| E09.10 \| E09.11 \| E09.21 \| E09.22 \| E09.29 \| E09.311 \| E09.319 \| E09.321 \| E09.3211 \| E09.3212 \| E09.3213 \| E09.3219 \| E09.329 \| E09.3291 \| E09.3292 \| E09.3293 \| E09.3299 \| E09.331 \| E09.3311 \| E09.3312 \| E09.3313 \| E09.3319 \| E09.339 \| E09.3391 \| E09.3392 \| E09.3393 \| E09.3399 \| E09.341 \| E09.3411 \| E09.3412 \| E09.3413 \| E09.3419 \| E09.349 \| E09.3491 \| E09.3492 \| E09.3493 \| E09.3499 \| E09.351 \| E09.3511 \| E09.3512 \| E09.3513 \| E09.3519 \| E09.3521 \| E09.3522 \| E09.3523 \| E09.3529 \| E09.3531 \| E09.3532 \| E09.3533 \| E09.3539 \| E09.3541 \| E09.3542 \| E09.3543 \| E09.3549 \| E09.3551 \| E09.3552 \| E09.3553 \| E09.3559 \| E09.359 \| E09.3591 \| E09.3592 \| E09.3593 \| E09.3599 \| E09.36 \| E09.37X1 \| E09.37X2 \| E09.37X3 \| E09.37X9 \| E09.39 \| E09.40 \| E09.41 \| E09.42 \| E09.43 \| E09.44 \| E09.49 \| E09.51 \| E09.52 \| E09.59 \| E09.610 \| E09.618 \| E09.620 \| E09.621 \| E09.622 \| E09.628 \| E09.630 \| E09.638 \| E09.641 \| E09.649 \| E09.65 \| E09.69 \| E09.8 \| E09.9 \| E10.10 \| E10.11 \| E10.21 \| E10.22 \| E10.29 \| E10.311 \| E10.319 \| E10.321 \| E10.3211 \| E10.3212 \| E10.3213 \| E10.3219 \| E10.329 \| E10.3291 \| E10.3292 \| E10.3293 \| E10.3299 \| E10.331 \| E10.3311 \| E10.3312 \| E10.3313 \| E10.3319 \| E10.339 \| E10.3391 \| E10.3392 \| E10.3393 \| E10.3399 \| E10.341 \| E10.3411 \| E10.3412 \| E10.3413 \| E10.3419 \| E10.349 \| E10.3491 \| E10.3492 \| E10.3493 \| E10.3499 \| E10.351 \| E10.3511 \| E10.3512 \| E10.3513 \| E10.3519 \| E10.3521 \| E10.3522 \| E10.3523 \| E10.3529 \| E10.3531 \| E10.3532 \| E10.3533 \| E10.3539 \| E10.3541 \| E10.3542 \| E10.3543 \| E10.3549 \| E10.3551 \| E10.3552 \| E10.3553 \| E10.3559 \| E10.359 \| E10.3591 \| E10.3592 \| E10.3593 \| E10.3599 \| E10.36 \| E10.37X1 \| E10.37X2 \| E10.37X3 \| E10.37X9 \| E10.39 \| E10.40 \| E10.41 \| E10.42 \| E10.43 \| E10.44 \| E10.49 \| E10.51 \| E10.52 \| E10.59 \| E10.610 \| E10.618 \| E10.620 \| E10.621 \| E10.622 \| E10.628 \| E10.630 \| E10.638 \| E10.641 \| E10.649 \| E10.65 \| E10.69 \| E10.8 \| E10.9 \| E11.00 \| E11.01 \| E11.10 \| E11.11 \| E11.21 \| E11.22 \| E11.29 \| E11.311 \| E11.319 \| E11.321 \| E11.3211 \| E11.3212 \| E11.3213 \| E11.3219 \| E11.329 \| E11.3291 \| E11.3292 \| E11.3293 \| E11.3299 \| E11.331 \| E11.3311 \| E11.3312 \| E11.3313 \| E11.3319 \| E11.339 \| E11.3391 \| E11.3392 \| E11.3393 \| E11.3399 \| E11.341 \| E11.3411 \| E11.3412 \| E11.3413 \| E11.3419 \| E11.349 \| E11.3491 \| E11.3492 \| E11.3493 \| E11.3499 \| E11.351 \| E11.3511 \| E11.3512 \| E11.3513 \| E11.3519 \| E11.3521 \| E11.3522 \| E11.3523 \| E11.3529 \| E11.3531 \| E11.3532 \| E11.3533 \| E11.3539 \| E11.3541 \| E11.3542 \| E11.3543 \| E11.3549 \| E11.3551 \| E11.3552 \| E11.3553 \| E11.3559 \| E11.359 \| E11.3591 \| E11.3592 \| E11.3593 \| E11.3599 \| E11.36 \| E11.37X1 \| E11.37X2 \| E11.37X3 \| E11.37X9 \| E11.39 \| E11.40 \| E11.41 \| E11.42 \| E11.43 \| E11.44 \| E11.49 \| E11.51 \| E11.52 \| E11.59 \| E11.610 \| E11.618 \| E11.620 \| E11.621 \| E11.622 \| E11.628 \| E11.630 \| E11.638 \| E11.641 \| E11.649 \| E11.65 \| E11.69 \| E11.8 \| E11.9 \| E13.00 \| E13.01 \| E13.10 \| E13.11 \| E13.21 \| E13.22 \| E13.29 \| E13.311 \| E13.319 \| E13.321 \| E13.3211 \| E13.3212 \| E13.3213 \| E13.3219 \| E13.329 \| E13.3291 \| E13.3292 \| E13.3293 \| E13.3299 \| E13.331 \| E13.3311 \| E13.3312 \| E13.3313 \| E13.3319 \| E13.339 \| E13.3391 \| E13.3392 \| E13.3393 \| E13.3399 \| E13.341 \| E13.3411 \| E13.3412 \| E13.3413 \| E13.3419 \| E13.349 \| E13.3491 \| E13.3492 \| E13.3493 \| E13.3499 \| E13.351 \| E13.3511 \| E13.3512 \| E13.3513 \| E13.3519 \| E13.3521 \| E13.3522 \| E13.3523 \| E13.3529 \| E13.3531 \| E13.3532 \| E13.3533 \| E13.3539 \| E13.3541 \| E13.3542 \| E13.3543 \| E13.3549 \| E13.3551 \| E13.3552 \| E13.3553 \| E13.3559 \| E13.359 \| E13.3591 \| E13.3592 \| E13.3593 \| E13.3599 \| E13.36 \| E13.39 \| E13.40 \| E13.41 \| E13.42 \| E13.43 \| E13.44 \| E13.49 \| E13.51 \| E13.52 \| E13.59 \| E13.610 \| E13.618 \| E13.620 \| E13.621 \| E13.622 \| E13.628 \| E13.630 \| E13.638 \| E13.641 \| E13.649 \| E13.65 \| E13.69 \| E13.8 \| E13.9 |
| Epilepsy | G40.001 \| G40.009 \| G40.011 \| G40.019 \| G40.101 \| G40.109 \| G40.111 \| G40.119 \| G40.201 \| G40.209 \| G40.211 \| G40.219 \| G40.301 \| G40.309 \| G40.311 \| G40.319 \| G40.401 \| G40.409 \| G40.411 \| G40.419 \| G40.42 \| G40.501 \| G40.509 \| G40.801 \| G40.802 \| G40.803 \| G40.804 \| G40.811 \| G40.812 \| G40.813 \| G40.814 \| G40.821 \| G40.822 \| G40.823 \| G40.824 \| G40.833 \| G40.834 \| G40.89 \| G40.901 \| G40.909 \| G40.911 \| G40.919 \| G40.A01 \| G40.A09 \| G40.A11 \| G40.A19 \| G40.B01 \| G40.B09 \| G40.B11 \| G40.B19 |
| Fibromyalgia, chronic pain and fatigue | G89.21 \| G89.22 \| G89.28 \| G89.29 \| G89.3 \| G89.4 \| M54.10 \| M54.11 \| M54.12 \| M54.13 \| M54.14 \| M54.15 \| M54.16 \| M54.17 \| M54.18 \| M60.80 \| M60.811 \| M60.812 \| M60.819 \| M60.821 \| M60.822 \| M60.829 \| M60.831 \| M60.832 \| M60.839 \| M60.841 \| M60.842 \| M60.849 \| M60.851 \| M60.852 \| M60.859 \| M60.861 \| M60.862 \| M60.869 \| M60.871 \| M60.872 \| M60.879 \| M60.88 \| M60.89 \| M60.9 \| M79.1 \| M79.10 \| M79.11 \| M79.12 \| M79.18 \| M79.2 \| M79.7 \| R53.82 |
| Glaucoma | H40.011 \| H40.012 \| H40.013 \| H40.019 \| H40.021 \| H40.022 \| H40.023 \| H40.029 \| H40.041 \| H40.042 \| H40.043 \| H40.049 \| H40.051 \| H40.052 \| H40.053 \| H40.059 \| H40.10X0 \| H40.10X1 \| H40.10X2 \| H40.10X3 \| H40.10X4 \| H40.1110 \| H40.1111 \| H40.1112 \| H40.1113 \| H40.1114 \| H40.1120 \| H40.1121 \| H40.1122 \| H40.1123 \| H40.1124 \| H40.1130 \| H40.1131 \| H40.1132 \| H40.1133 \| H40.1134 \| H40.1190 \| H40.1191 \| H40.1192 \| H40.1193 \| H40.1194 \| H40.11X0 \| H40.11X1 \| H40.11X2 \| H40.11X3 \| H40.11X4 \| H40.1210 \| H40.1211 \| H40.1212 \| H40.1213 \| H40.1214 \| H40.1220 \| H40.1221 \| H40.1222 \| H40.1223 \| H40.1224 \| H40.1230 \| H40.1231 \| H40.1232 \| H40.1233 \| H40.1234 \| H40.1290 \| H40.1291 \| H40.1292 \| H40.1293 \| H40.1294 \| H40.1310 \| H40.1311 \| H40.1312 \| H40.1313 \| H40.1314 \| H40.1320 \| H40.1321 \| H40.1322 \| H40.1323 \| H40.1324 \| H40.1330 \| H40.1331 \| H40.1332 \| H40.1333 \| H40.1334 \| H40.1390 \| H40.1391 \| H40.1392 \| H40.1393 \| H40.1394 \| H40.1410 \| H40.1411 \| H40.1412 \| H40.1413 \| H40.1414 \| H40.1420 \| H40.1421 \| H40.1422 \| H40.1423 \| H40.1424 \| H40.1430 \| H40.1431 \| H40.1432 \| H40.1433 \| H40.1434 \| H40.1490 \| H40.1491 \| H40.1492 \| H40.1493 \| H40.1494 \| H40.151 \| H40.152 \| H40.153 \| H40.159 \| H40.20X0 \| H40.20X1 \| H40.20X2 \| H40.20X3 \| H40.20X4 \| H40.211 \| H40.212 \| H40.213 \| H40.219 \| H40.2210 \| H40.2211 \| H40.2212 \| H40.2213 \| H40.2214 \| H40.2220 \| H40.2221 \| H40.2222 \| H40.2223 \| H40.2224 \| H40.2230 \| H40.2231 \| H40.2232 \| H40.2233 \| H40.2234 \| H40.2290 \| H40.2291 \| H40.2292 \| H40.2293 \| H40.2294 \| H40.231 \| H40.232 \| H40.233 \| H40.239 \| H40.241 \| H40.242 \| H40.243 \| H40.249 \| H40.30X0 \| H40.30X1 \| H40.30X2 \| H40.30X3 \| H40.30X4 \| H40.31X0 \| H40.31X1 \| H40.31X2 \| H40.31X3 \| H40.31X4 \| H40.32X0 \| H40.32X1 \| H40.32X2 \| H40.32X3 \| H40.32X4 \| H40.33X0 \| H40.33X1 \| H40.33X2 \| H40.33X3 \| H40.33X4 \| H40.40X0 \| H40.40X1 \| H40.40X2 \| H40.40X3 \| H40.40X4 \| H40.41X0 \| H40.41X1 \| H40.41X2 \| H40.41X3 \| H40.41X4 \| H40.42X0 \| H40.42X1 \| H40.42X2 \| H40.42X3 \| H40.42X4 \| H40.43X0 \| H40.43X1 \| H40.43X2 \| H40.43X3 \| H40.43X4 \| H40.50X0 \| H40.50X1 \| H40.50X2 \| H40.50X3 \| H40.50X4 \| H40.51X0 \| H40.51X1 \| H40.51X2 \| H40.51X3 \| H40.51X4 \| H40.52X0 \| H40.52X1 \| H40.52X2 \| H40.52X3 \| H40.52X4 \| H40.53X0 \| H40.53X1 \| H40.53X2 \| H40.53X3 \| H40.53X4 \| H40.60X0 \| H40.60X1 \| H40.60X2 \| H40.60X3 \| H40.60X4 \| H40.61X0 \| H40.61X1 \| H40.61X2 \| H40.61X3 \| H40.61X4 \| H40.62X0 \| H40.62X1 \| H40.62X2 \| H40.62X3 \| H40.62X4 \| H40.63X0 \| H40.63X1 \| H40.63X2 \| H40.63X3 \| H40.63X4 \| H40.811 \| H40.812 \| H40.813 \| H40.819 \| H40.821 \| H40.822 \| H40.823 \| H40.829 \| H40.831 \| H40.832 \| H40.833 \| H40.839 \| H40.89 \| H40.9 \| H42 \| H44.511 \| H44.512 \| H44.513 \| H44.519 \| H47.231 \| H47.232 \| H47.233 \| H47.239 \| Q15.0 |
| Heart failure and non-ischemic heart disease | I09.81 \| I42.0 \| I42.5 \| I42.6 \| I42.7 \| I42.8 \| I43 \| I50.1 \| I50.20 \| I50.21 \| I50.22 \| I50.23 \| I50.30 \| I50.31 \| I50.32 \| I50.33 \| I50.40 \| I50.41 \| I50.42 \| I50.43 \| I50.810 \| I50.811 \| I50.812 \| I50.813 \| I50.814 \| I50.82 \| I50.83 \| I50.84 \| I50.89 \| I50.9 \| P29.0 |
| Hip/pelvic fracture | M84.350A \| M84.351A \| M84.352A \| M84.353A \| M84.359A \| M84.451A \| M84.452A \| M84.453A \| M84.459A \| M84.550A \| M84.551A \| M84.552A \| M84.553A \| M84.559A \| M84.650A \| M84.651A \| M84.652A \| M84.653A \| M84.659A \| M97.01XA \| M97.02XA \| S32.301A \| S32.301B \| S32.302A \| S32.302B \| S32.309A \| S32.309B \| S32.311A \| S32.311B \| S32.312A \| S32.312B \| S32.313A \| S32.313B \| S32.314A \| S32.314B \| S32.315A \| S32.315B \| S32.316A \| S32.316B \| S32.391A \| S32.391B \| S32.392A \| S32.392B \| S32.399A \| S32.399B \| S32.401A \| S32.401B \| S32.402A \| S32.402B \| S32.409A \| S32.409B \| S32.411A \| S32.411B \| S32.412A \| S32.412B \| S32.413A \| S32.413B \| S32.414A \| S32.414B \| S32.415A \| S32.415B \| S32.416A \| S32.416B \| S32.421A \| S32.421B \| S32.422A \| S32.422B \| S32.423A \| S32.423B \| S32.424A \| S32.424B \| S32.425A \| S32.425B \| S32.426A \| S32.426B \| S32.431A \| S32.431B \| S32.432A \| S32.432B \| S32.433A \| S32.433B \| S32.434A \| S32.434B \| S32.435A \| S32.435B \| S32.436A \| S32.436B \| S32.441A \| S32.441B \| S32.442A \| S32.442B \| S32.443A \| S32.443B \| S32.444A \| S32.444B \| S32.445A \| S32.445B \| S32.446A \| S32.446B \| S32.451A \| S32.451B \| S32.452A \| S32.452B \| S32.453A \| S32.453B \| S32.454A \| S32.454B \| S32.455A \| S32.455B \| S32.456A \| S32.456B \| S32.461A \| S32.461B \| S32.462A \| S32.462B \| S32.463A \| S32.463B \| S32.464A \| S32.464B \| S32.465A \| S32.465B \| S32.466A \| S32.466B \| S32.471A \| S32.471B \| S32.472A \| S32.472B \| S32.473A \| S32.473B \| S32.474A \| S32.474B \| S32.475A \| S32.475B \| S32.476A \| S32.476B \| S32.481A \| S32.481B \| S32.482A \| S32.482B \| S32.483A \| S32.483B \| S32.484A \| S32.484B \| S32.485A \| S32.485B \| S32.486A \| S32.486B \| S32.491A \| S32.491B \| S32.492A \| S32.492B \| S32.499A \| S32.499B \| S32.501A \| S32.501B \| S32.502A \| S32.502B \| S32.509A \| S32.509B \| S32.511A \| S32.511B \| S32.512A \| S32.512B \| S32.519A \| S32.519B \| S32.591A \| S32.591B \| S32.592A \| S32.592B \| S32.599A \| S32.599B \| S32.601A \| S32.601B \| S32.602A \| S32.602B \| S32.609A \| S32.609B \| S32.611A \| S32.611B \| S32.612A \| S32.612B \| S32.613A \| S32.613B \| S32.614A \| S32.614B \| S32.615A \| S32.615B \| S32.616A \| S32.616B \| S32.691A \| S32.691B \| S32.692A \| S32.692B \| S32.699A \| S32.699B \| S32.810A \| S32.810B \| S32.811A \| S32.811B \| S32.82XA \| S32.82XB \| S32.89XA \| S32.89XB \| S32.9XXA \| S32.9XXB \| S72.001A \| S72.001B \| S72.001C \| S72.002A \| S72.002B \| S72.002C \| S72.009A \| S72.009B \| S72.009C \| S72.011A \| S72.011B \| S72.011C \| S72.012A \| S72.012B \| S72.012C \| S72.019A \| S72.019B \| S72.019C \| S72.021A \| S72.021B \| S72.021C \| S72.022A \| S72.022B \| S72.022C \| S72.023A \| S72.023B \| S72.023C \| S72.024A \| S72.024B \| S72.024C \| S72.025A \| S72.025B \| S72.025C \| S72.026A \| S72.026B \| S72.026C \| S72.031A \| S72.031B \| S72.031C \| S72.032A \| S72.032B \| S72.032C \| S72.033A \| S72.033B \| S72.033C \| S72.034A \| S72.034B \| S72.034C \| S72.035A \| S72.035B \| S72.035C \| S72.036A \| S72.036B \| S72.036C \| S72.041A \| S72.041B \| S72.041C \| S72.042A \| S72.042B \| S72.042C \| S72.043A \| S72.043B \| S72.043C \| S72.044A \| S72.044B \| S72.044C \| S72.045A \| S72.045B \| S72.045C \| S72.046A \| S72.046B \| S72.046C \| S72.051A \| S72.051B \| S72.051C \| S72.052A \| S72.052B \| S72.052C \| S72.059A \| S72.059B \| S72.059C \| S72.061A \| S72.061B \| S72.061C \| S72.062A \| S72.062B \| S72.062C \| S72.063A \| S72.063B \| S72.063C \| S72.064A \| S72.064B \| S72.064C \| S72.065A \| S72.065B \| S72.065C \| S72.066A \| S72.066B \| S72.066C \| S72.091A \| S72.091B \| S72.091C \| S72.092A \| S72.092B \| S72.092C \| S72.099A \| S72.099B \| S72.099C \| S72.101A \| S72.101B \| S72.101C \| S72.102A \| S72.102B \| S72.102C \| S72.109A \| S72.109B \| S72.109C \| S72.111A \| S72.111B \| S72.111C \| S72.112A \| S72.112B \| S72.112C \| S72.113A \| S72.113B \| S72.113C \| S72.114A \| S72.114B \| S72.114C \| S72.115A \| S72.115B \| S72.115C \| S72.116A \| S72.116B \| S72.116C \| S72.121A \| S72.121B \| S72.121C \| S72.122A \| S72.122B \| S72.122C \| S72.123A \| S72.123B \| S72.123C \| S72.124A \| S72.124B \| S72.124C \| S72.125A \| S72.125B \| S72.125C \| S72.126A \| S72.126B \| S72.126C \| S72.131A \| S72.131B \| S72.131C \| S72.132A \| S72.132B \| S72.132C \| S72.133A \| S72.133B \| S72.133C \| S72.134A \| S72.134B \| S72.134C \| S72.135A \| S72.135B \| S72.135C \| S72.136A \| S72.136B \| S72.136C \| S72.141A \| S72.141B \| S72.141C \| S72.142A \| S72.142B \| S72.142C \| S72.143A \| S72.143B \| S72.143C \| S72.144A \| S72.144B \| S72.144C \| S72.145A \| S72.145B \| S72.145C \| S72.146A \| S72.146B \| S72.146C \| S72.21XA \| S72.21XB \| S72.21XC \| S72.22XA \| S72.22XB \| S72.22XC \| S72.23XA \| S72.23XB \| S72.23XC \| S72.24XA \| S72.24XB \| S72.24XC \| S72.25XA \| S72.25XB \| S72.25XC \| S72.26XA \| S72.26XB \| S72.26XC \| S79.001A \| S79.002A \| S79.009A \| S79.011A \| S79.012A \| S79.019A \| S79.091A \| S79.092A \| S79.099A |
| HIV/AIDS | B20 \| B97.35 \| Z21 |
| Hyperlipidemia | E78.0 \| E78.00 \| E78.01 \| E78.1 \| E78.2 \| E78.3 \| E78.4 \| E78.41 \| E78.49 \| E78.5 |
| Hypertension | H35.031 \| H35.032 \| H35.033 \| H35.039 \| I10 \| I11.0 \| I11.9 \| I15.0 \| I15.1 \| I15.2 \| I15.8 \| I15.9 \| I67.4 \| N26.2 |
| Hypothyroidism | E00.0 \| E00.1 \| E00.2 \| E00.9 \| E01.8 \| E02 \| E03.0 \| E03.1 \| E03.2 \| E03.3 \| E03.4 \| E03.8 \| E03.9 \| E89.0 |
| Intellectual disabilities and related conditions | E78.71 \| E78.72 \| F70 \| F71 \| F72 \| F73 \| F78 \| F78.A1 \| F78.A9 \| F79 \| P04.3 \| P04.41 \| P04.49 \| P96.1 \| P96.2 \| Q86.0 \| Q87.1 \| Q87.11 \| Q87.19 \| Q87.2 \| Q87.3 \| Q87.5 \| Q87.81 \| Q87.89 \| Q89.7 \| Q89.8 \| Q90.0 \| Q90.1 \| Q90.2 \| Q90.9 \| Q91.0 \| Q91.1 \| Q91.2 \| Q91.3 \| Q91.4 \| Q91.5 \| Q91.6 \| Q91.7 \| Q92.0 \| Q92.1 \| Q92.2 \| Q92.5 \| Q92.61 \| Q92.62 \| Q92.7 \| Q92.8 \| Q92.9 \| Q93.0 \| Q93.1 \| Q93.2 \| Q93.3 \| Q93.4 \| Q93.5 \| Q93.51 \| Q93.59 \| Q93.7 \| Q93.81 \| Q93.88 \| Q93.89 \| Q93.9 \| Q95.2 \| Q95.3 \| Q99.2 |
| Ischemic heart disease | I20.0 \| I20.1 \| I20.2 \| I20.8 \| I24.0 \| I24.1 \| I24.8 \| I25.10 \| I25.110 \| I25.111 \| I25.112 \| I25.118 \| I25.119 \| I25.3 \| I25.41 \| I25.42 \| I25.5 \| I25.6 \| I25.700 \| I25.701 \| I25.702 \| I25.708 \| I25.710 \| I25.711 \| I25.712 \| I25.718 \| I25.719 \| I25.720 \| I25.721 \| I25.722 \| I25.728 \| I25.729 \| I25.730 \| I25.731 \| I25.732 \| I25.738 \| I25.739 \| I25.750 \| I25.751 \| I25.752 \| I25.758 \| I25.759 \| I25.760 \| I25.761 \| I25.762 \| I25.768 \| I25.769 \| I25.790 \| I25.791 \| I25.792 \| I25.798 \| I25.799 \| I25.810 \| I25.811 \| I25.812 \| I25.82 \| I25.83 \| I25.84 \| I25.89 \| I25.9 |
| Learning disabilities and developmental delays^3^ | F80.0 \| F80.1 \| F80.2 \| F80.4 \| F80.81 \| F80.82 \| F80.89 \| F80.9 \| F81.0 \| F81.2 \| F81.81 \| F81.89 \| F81.9 \| F82 \| F88 \| F89 \| H93.25 \| R48.0 |
| Leukemias and lymphomas | C81.00 \| C81.01 \| C81.02 \| C81.03 \| C81.04 \| C81.05 \| C81.06 \| C81.07 \| C81.08 \| C81.09 \| C81.10 \| C81.11 \| C81.12 \| C81.13 \| C81.14 \| C81.15 \| C81.16 \| C81.17 \| C81.18 \| C81.19 \| C81.20 \| C81.21 \| C81.22 \| C81.23 \| C81.24 \| C81.25 \| C81.26 \| C81.27 \| C81.28 \| C81.29 \| C81.30 \| C81.31 \| C81.32 \| C81.33 \| C81.34 \| C81.35 \| C81.36 \| C81.37 \| C81.38 \| C81.39 \| C81.40 \| C81.41 \| C81.42 \| C81.43 \| C81.44 \| C81.45 \| C81.46 \| C81.47 \| C81.48 \| C81.49 \| C81.70 \| C81.71 \| C81.72 \| C81.73 \| C81.74 \| C81.75 \| C81.76 \| C81.77 \| C81.78 \| C81.79 \| C81.90 \| C81.91 \| C81.92 \| C81.93 \| C81.94 \| C81.95 \| C81.96 \| C81.97 \| C81.98 \| C81.99 \| C82.00 \| C82.01 \| C82.02 \| C82.03 \| C82.04 \| C82.05 \| C82.06 \| C82.07 \| C82.08 \| C82.09 \| C82.10 \| C82.11 \| C82.12 \| C82.13 \| C82.14 \| C82.15 \| C82.16 \| C82.17 \| C82.18 \| C82.19 \| C82.20 \| C82.21 \| C82.22 \| C82.23 \| C82.24 \| C82.25 \| C82.26 \| C82.27 \| C82.28 \| C82.29 \| C82.30 \| C82.31 \| C82.32 \| C82.33 \| C82.34 \| C82.35 \| C82.36 \| C82.37 \| C82.38 \| C82.39 \| C82.40 \| C82.41 \| C82.42 \| C82.43 \| C82.44 \| C82.45 \| C82.46 \| C82.47 \| C82.48 \| C82.49 \| C82.50 \| C82.51 \| C82.52 \| C82.53 \| C82.54 \| C82.55 \| C82.56 \| C82.57 \| C82.58 \| C82.59 \| C82.60 \| C82.61 \| C82.62 \| C82.63 \| C82.64 \| C82.65 \| C82.66 \| C82.67 \| C82.68 \| C82.69 \| C82.80 \| C82.81 \| C82.82 \| C82.83 \| C82.84 \| C82.85 \| C82.86 \| C82.87 \| C82.88 \| C82.89 \| C82.90 \| C82.91 \| C82.92 \| C82.93 \| C82.94 \| C82.95 \| C82.96 \| C82.97 \| C82.98 \| C82.99 \| C83.00 \| C83.01 \| C83.02 \| C83.03 \| C83.04 \| C83.05 \| C83.06 \| C83.07 \| C83.08 \| C83.09 \| C83.10 \| C83.11 \| C83.12 \| C83.13 \| C83.14 \| C83.15 \| C83.16 \| C83.17 \| C83.18 \| C83.19 \| C83.30 \| C83.31 \| C83.32 \| C83.33 \| C83.34 \| C83.35 \| C83.36 \| C83.37 \| C83.38 \| C83.39 \| C83.50 \| C83.51 \| C83.52 \| C83.53 \| C83.54 \| C83.55 \| C83.56 \| C83.57 \| C83.58 \| C83.59 \| C83.70 \| C83.71 \| C83.72 \| C83.73 \| C83.74 \| C83.75 \| C83.76 \| C83.77 \| C83.78 \| C83.79 \| C83.80 \| C83.81 \| C83.82 \| C83.83 \| C83.84 \| C83.85 \| C83.86 \| C83.87 \| C83.88 \| C83.89 \| C83.90 \| C83.91 \| C83.92 \| C83.93 \| C83.94 \| C83.95 \| C83.96 \| C83.97 \| C83.98 \| C83.99 \| C84.00 \| C84.01 \| C84.02 \| C84.03 \| C84.04 \| C84.05 \| C84.06 \| C84.07 \| C84.08 \| C84.09 \| C84.10 \| C84.11 \| C84.12 \| C84.13 \| C84.14 \| C84.15 \| C84.16 \| C84.17 \| C84.18 \| C84.19 \| C84.40 \| C84.41 \| C84.42 \| C84.43 \| C84.44 \| C84.45 \| C84.46 \| C84.47 \| C84.48 \| C84.49 \| C84.60 \| C84.61 \| C84.62 \| C84.63 \| C84.64 \| C84.65 \| C84.66 \| C84.67 \| C84.68 \| C84.69 \| C84.70 \| C84.71 \| C84.72 \| C84.73 \| C84.74 \| C84.75 \| C84.76 \| C84.77 \| C84.78 \| C84.79 \| C84.7A \| C84.90 \| C84.91 \| C84.92 \| C84.93 \| C84.94 \| C84.95 \| C84.96 \| C84.97 \| C84.98 \| C84.99 \| C84.A0 \| C84.A1 \| C84.A2 \| C84.A3 \| C84.A4 \| C84.A5 \| C84.A6 \| C84.A7 \| C84.A8 \| C84.A9 \| C84.Z0 \| C84.Z1 \| C84.Z2 \| C84.Z3 \| C84.Z4 \| C84.Z5 \| C84.Z6 \| C84.Z7 \| C84.Z8 \| C84.Z9 \| C85.10 \| C85.11 \| C85.12 \| C85.13 \| C85.14 \| C85.15 \| C85.16 \| C85.17 \| C85.18 \| C85.19 \| C85.20 \| C85.21 \| C85.22 \| C85.23 \| C85.24 \| C85.25 \| C85.26 \| C85.27 \| C85.28 \| C85.29 \| C85.80 \| C85.81 \| C85.82 \| C85.83 \| C85.84 \| C85.85 \| C85.86 \| C85.87 \| C85.88 \| C85.89 \| C85.90 \| C85.91 \| C85.92 \| C85.93 \| C85.94 \| C85.95 \| C85.96 \| C85.97 \| C85.98 \| C85.99 \| C86.0 \| C86.1 \| C86.2 \| C86.3 \| C86.4 \| C86.5 \| C86.6 \| C88.4 \| C90.10 \| C90.11 \| C90.12 \| C91.00 \| C91.01 \| C91.02 \| C91.10 \| C91.11 \| C91.12 \| C91.30 \| C91.31 \| C91.32 \| C91.40 \| C91.41 \| C91.42 \| C91.50 \| C91.51 \| C91.52 \| C91.60 \| C91.61 \| C91.62 \| C91.90 \| C91.91 \| C91.92 \| C91.A0 \| C91.A1 \| C91.A2 \| C91.Z0 \| C91.Z1 \| C91.Z2 \| C92.00 \| C92.01 \| C92.02 \| C92.10 \| C92.11 \| C92.12 \| C92.20 \| C92.21 \| C92.22 \| C92.30 \| C92.31 \| C92.32 \| C92.40 \| C92.41 \| C92.42 \| C92.50 \| C92.51 \| C92.52 \| C92.60 \| C92.61 \| C92.62 \| C92.90 \| C92.91 \| C92.92 \| C92.A0 \| C92.A1 \| C92.A2 \| C92.Z0 \| C92.Z1 \| C92.Z2 \| C93.00 \| C93.01 \| C93.02 \| C93.10 \| C93.11 \| C93.12 \| C93.30 \| C93.31 \| C93.32 \| C93.90 \| C93.91 \| C93.92 \| C93.Z0 \| C93.Z1 \| C93.Z2 \| C94.00 \| C94.01 \| C94.02 \| C94.20 \| C94.21 \| C94.22 \| C94.30 \| C94.31 \| C94.32 \| C94.80 \| C94.81 \| C94.82 \| C95.00 \| C95.01 \| C95.02 \| C95.10 \| C95.11 \| C95.12 \| C95.90 \| C95.91 \| C95.92 \| C96.4 \| C96.9 \| C96.Z \| D45 \| Z85.6 \| Z85.71 \| Z85.72 \| Z85.79 |
| Liver disease, cirrhosis and other liver conditions | K70.0 \| K70.10 \| K70.11 \| K70.2 \| K70.30 \| K70.31 \| K70.40 \| K70.41 \| K70.9 \| K71.0 \| K71.11 \| K71.7 \| K71.8 \| K71.9 \| K72.00 \| K72.01 \| K72.10 \| K72.11 \| K72.90 \| K72.91 \| K74.0 \| K74.00 \| K74.01 \| K74.02 \| K74.1 \| K74.2 \| K74.3 \| K74.4 \| K74.5 \| K74.60 \| K74.69 \| K75.0 \| K75.1 \| K75.81 \| K75.89 \| K75.9 \| K76.0 \| K76.1 \| K76.2 \| K76.3 \| K76.5 \| K76.6 \| K76.7 \| K76.81 \| K76.82 \| K76.89 \| K76.9 \| K77 \| K80.30 \| K80.31 \| K80.32 \| K80.33 \| K80.34 \| K80.35 \| K80.36 \| K80.37 \| K83.0 \| R16.0 \| R16.2 \| Z48.23 \| Z94.4 |
| Migraine and chronic headache | G43.001 \| G43.009 \| G43.011 \| G43.019 \| G43.101 \| G43.109 \| G43.111 \| G43.119 \| G43.401 \| G43.409 \| G43.411 \| G43.419 \| G43.501 \| G43.509 \| G43.511 \| G43.519 \| G43.601 \| G43.609 \| G43.611 \| G43.619 \| G43.701 \| G43.709 \| G43.711 \| G43.719 \| G43.801 \| G43.809 \| G43.811 \| G43.819 \| G43.821 \| G43.829 \| G43.831 \| G43.839 \| G43.901 \| G43.909 \| G43.911 \| G43.919 \| G43.A0 \| G43.A1 \| G43.B0 \| G43.B1 \| G43.C0 \| G43.C1 \| G43.D0 \| G43.D1 \| G44.001 \| G44.009 \| G44.011 \| G44.019 \| G44.021 \| G44.029 \| G44.031 \| G44.039 \| G44.041 \| G44.049 \| G44.051 \| G44.059 \| G44.091 \| G44.099 \| G44.1 \| G44.201 \| G44.209 \| G44.211 \| G44.219 \| G44.221 \| G44.229 \| G44.301 \| G44.309 \| G44.311 \| G44.319 \| G44.321 \| G44.329 \| G44.40 \| G44.41 \| G44.51 \| G44.52 \| G44.53 \| G44.59 \| G44.81 \| G44.82 \| G44.83 \| G44.84 \| G44.85 \| G44.86 \| G44.89 |
| Mobility impairments | G04.1 \| G11.4 \| G81.00 \| G81.01 \| G81.02 \| G81.03 \| G81.04 \| G81.10 \| G81.11 \| G81.12 \| G81.13 \| G81.14 \| G81.90 \| G81.91 \| G81.92 \| G81.93 \| G81.94 \| G82.20 \| G82.21 \| G82.22 \| G82.50 \| G82.51 \| G82.52 \| G82.53 \| G82.54 \| G83.0 \| G83.10 \| G83.11 \| G83.12 \| G83.13 \| G83.14 \| G83.20 \| G83.21 \| G83.22 \| G83.23 \| G83.24 \| G83.30 \| G83.31 \| G83.32 \| G83.33 \| G83.34 \| G83.4 \| G83.5 \| G83.81 \| G83.82 \| G83.83 \| G83.84 \| G83.89 \| G83.9 \| I69.031 \| I69.032 \| I69.033 \| I69.034 \| I69.039 \| I69.041 \| I69.042 \| I69.043 \| I69.044 \| I69.049 \| I69.051 \| I69.052 \| I69.053 \| I69.054 \| I69.059 \| I69.061 \| I69.062 \| I69.063 \| I69.064 \| I69.065 \| I69.069 \| I69.131 \| I69.132 \| I69.133 \| I69.134 \| I69.139 \| I69.141 \| I69.142 \| I69.143 \| I69.144 \| I69.149 \| I69.151 \| I69.152 \| I69.153 \| I69.154 \| I69.159 \| I69.161 \| I69.162 \| I69.163 \| I69.164 \| I69.165 \| I69.169 \| I69.231 \| I69.232 \| I69.233 \| I69.234 \| I69.239 \| I69.241 \| I69.242 \| I69.243 \| I69.244 \| I69.249 \| I69.251 \| I69.252 \| I69.253 \| I69.254 \| I69.259 \| I69.261 \| I69.262 \| I69.263 \| I69.264 \| I69.265 \| I69.269 \| I69.331 \| I69.332 \| I69.333 \| I69.334 \| I69.339 \| I69.341 \| I69.342 \| I69.343 \| I69.344 \| I69.349 \| I69.351 \| I69.352 \| I69.353 \| I69.354 \| I69.359 \| I69.361 \| I69.362 \| I69.363 \| I69.364 \| I69.365 \| I69.369 \| I69.831 \| I69.832 \| I69.833 \| I69.834 \| I69.839 \| I69.841 \| I69.842 \| I69.843 \| I69.844 \| I69.849 \| I69.851 \| I69.852 \| I69.853 \| I69.854 \| I69.859 \| I69.861 \| I69.862 \| I69.863 \| I69.864 \| I69.865 \| I69.869 \| I69.931 \| I69.932 \| I69.933 \| I69.934 \| I69.939 \| I69.941 \| I69.942 \| I69.943 \| I69.944 \| I69.949 \| I69.951 \| I69.952 \| I69.953 \| I69.954 \| I69.959 \| I69.961 \| I69.962 \| I69.963 \| I69.964 \| I69.965 \| I69.969 |
| Multiple sclerosis and transverse myelitis | G35 \| G36.0 \| G36.1 \| G36.8 \| G36.9 \| G37.1 \| G37.2 \| G37.3 \| G37.4 \| G37.8 \| G37.9 |
| Muscular dystrophy | G71.0 \| G71.00 \| G71.01 \| G71.02 \| G71.031 \| G71.032 \| G71.033 \| G71.0340 \| G71.0341 \| G71.0342 \| G71.0349 \| G71.035 \| G71.038 \| G71.039 \| G71.09 \| G71.11 \| G71.2 \| G71.20 \| G71.21 \| G71.220 \| G71.228 \| G71.29 |
| Non-Alzheimer’s dementia | F01.50 \| F01.51 \| F01.511 \| F01.518 \| F01.52 \| F01.53 \| F01.54 \| F01.A0 \| F01.A11 \| F01.A18 \| F01.A2 \| F01.A3 \| F01.A4 \| F01.B0 \| F01.B11 \| F01.B18 \| F01.B2 \| F01.B3 \| F01.B4 \| F01.C0 \| F01.C11 \| F01.C18 \| F01.C2 \| F01.C3 \| F01.C4 \| F02.80 \| F02.81 \| F02.811 \| F02.818 \| F02.82 \| F02.83 \| F02.84 \| F02.A0 \| F02.A11 \| F02.A18 \| F02.A2 \| F02.A3 \| F02.A4 \| F02.B0 \| F02.B11 \| F02.B18 \| F02.B2 \| F02.B3 \| F02.B4 \| F02.C0 \| F02.C11 \| F02.C18 \| F02.C2 \| F02.C3 \| F02.C4 \| F03.90 \| F03.91 \| F03.911 \| F03.918 \| F03.92 \| F03.93 \| F03.94 \| F03.A0 \| F03.A11 \| F03.A18 \| F03.A2 \| F03.A3 \| F03.A4 \| F03.B0 \| F03.B11 \| F03.B18 \| F03.B2 \| F03.B3 \| F03.B4 \| F03.C0 \| F03.C11 \| F03.C18 \| F03.C2 \| F03.C3 \| F03.C4 \| F05 \| G13.8 \| G31.01 \| G31.09 \| G31.1 \| G31.2 \| G31.83 \| G94 \| R41.81 |
| Obesity | E66.01 \| E66.09 \| E66.1 \| E66.2 \| E66.8 \| E66.9 \| Z68.30 \| Z68.31 \| Z68.32 \| Z68.33 \| Z68.34 \| Z68.35 \| Z68.36 \| Z68.37 \| Z68.38 \| Z68.39 \| Z68.41 \| Z68.42 \| Z68.43 \| Z68.44 \| Z68.45 |
| Osteoporosis | M80.00XA \| M80.011A \| M80.012A \| M80.019A \| M80.021A \| M80.022A \| M80.029A \| M80.031A \| M80.032A \| M80.039A \| M80.041A \| M80.042A \| M80.049A \| M80.051A \| M80.052A \| M80.059A \| M80.061A \| M80.062A \| M80.069A \| M80.071A \| M80.072A \| M80.079A \| M80.08XA \| M80.0AXA \| M80.80XA \| M80.811A \| M80.812A \| M80.819A \| M80.821A \| M80.822A \| M80.829A \| M80.831A \| M80.832A \| M80.839A \| M80.841A \| M80.842A \| M80.849A \| M80.851A \| M80.852A \| M80.859A \| M80.861A \| M80.862A \| M80.869A \| M80.871A \| M80.872A \| M80.879A \| M80.88XA \| M80.8AXA \| M81.0 \| M81.6 \| M81.8 |
| Parkinson’s disease and secondary parkinsonism | G20 \| G21.11 \| G21.19 \| G21.3 \| G21.4 \| G21.8 \| G21.9 |
| Peripheral vascular disease | I70.0 \| I70.1 \| I70.201 \| I70.202 \| I70.203 \| I70.208 \| I70.209 \| I70.211 \| I70.212 \| I70.213 \| I70.218 \| I70.219 \| I70.221 \| I70.222 \| I70.223 \| I70.228 \| I70.229 \| I70.231 \| I70.232 \| I70.233 \| I70.234 \| I70.235 \| I70.238 \| I70.239 \| I70.241 \| I70.242 \| I70.243 \| I70.244 \| I70.245 \| I70.248 \| I70.249 \| I70.25 \| I70.291 \| I70.292 \| I70.293 \| I70.298 \| I70.299 \| I70.331 \| I70.332 \| I70.333 \| I70.334 \| I70.335 \| I70.338 \| I70.339 \| I70.341 \| I70.342 \| I70.343 \| I70.344 \| I70.345 \| I70.348 \| I70.349 \| I70.35 \| I70.431 \| I70.432 \| I70.433 \| I70.434 \| I70.435 \| I70.438 \| I70.439 \| I70.441 \| I70.442 \| I70.443 \| I70.444 \| I70.445 \| I70.448 \| I70.449 \| I70.45 \| I70.531 \| I70.532 \| I70.533 \| I70.534 \| I70.535 \| I70.538 \| I70.539 \| I70.541 \| I70.542 \| I70.543 \| I70.544 \| I70.545 \| I70.548 \| I70.549 \| I70.55 \| I70.631 \| I70.632 \| I70.633 \| I70.634 \| I70.635 \| I70.638 \| I70.639 \| I70.641 \| I70.642 \| I70.643 \| I70.644 \| I70.645 \| I70.648 \| I70.649 \| I70.65 \| I70.731 \| I70.732 \| I70.733 \| I70.734 \| I70.735 \| I70.738 \| I70.739 \| I70.741 \| I70.742 \| I70.743 \| I70.744 \| I70.745 \| I70.748 \| I70.749 \| I70.75 \| I70.92 \| I73.81 \| I73.89 \| I73.9 \| I79.1 \| I79.8 |
| Personality disorders | F21 \| F34.0 \| F60.0 \| F60.1 \| F60.2 \| F60.3 \| F60.4 \| F60.5 \| F60.6 \| F60.7 \| F60.81 \| F60.89 \| F60.9 \| F68.10 \| F68.11 \| F68.12 \| F68.13 \| F69 |
| Pneumonia | A01.03 \| A02.22 \| A06.5 \| A20.2 \| A21.2 \| A22.1 \| A31.0 \| A37.01 \| A37.11 \| A37.81 \| A37.91 \| A40.3 \| A42.0 \| A43.0 \| A48.1 \| A50.04 \| A54.84 \| B01.2 \| B05.2 \| B06.81 \| B37.1 \| B38.0 \| B38.2 \| B39.0 \| B39.2 \| B40.0 \| B40.2 \| B41.0 \| B58.3 \| B59 \| B66.4 \| B67.1 \| B77.81 \| B95.3 \| B96.0 \| B96.1 \| J09.X1 \| J10.00 \| J10.01 \| J10.08 \| J11.00 \| J11.08 \| J12.0 \| J12.1 \| J12.2 \| J12.3 \| J12.81 \| J12.82 \| J12.89 \| J12.9 \| J13 \| J14 \| J15.0 \| J15.1 \| J15.20 \| J15.211 \| J15.212 \| J15.29 \| J15.3 \| J15.4 \| J15.5 \| J15.6 \| J15.7 \| J15.8 \| J15.9 \| J16.0 \| J16.8 \| J17 \| J18.0 \| J18.1 \| J18.2 \| J18.8 \| J18.9 \| J20.0 \| J84.111 \| J84.116 \| J84.117 \| J84.178 \| J84.2 \| J85.1 \| J95.851 \| P23.0 \| P23.1 \| P23.2 \| P23.3 \| P23.4 \| P23.5 \| P23.6 \| P23.8 \| P23.9 \| Z87.01 |
| Pressure and chronic ulcers | L89.000 \| L89.001 \| L89.002 \| L89.003 \| L89.004 \| L89.006 \| L89.009 \| L89.010 \| L89.011 \| L89.012 \| L89.013 \| L89.014 \| L89.016 \| L89.019 \| L89.020 \| L89.021 \| L89.022 \| L89.023 \| L89.024 \| L89.026 \| L89.029 \| L89.100 \| L89.101 \| L89.102 \| L89.103 \| L89.104 \| L89.106 \| L89.109 \| L89.110 \| L89.111 \| L89.112 \| L89.113 \| L89.114 \| L89.116 \| L89.119 \| L89.120 \| L89.121 \| L89.122 \| L89.123 \| L89.124 \| L89.126 \| L89.129 \| L89.130 \| L89.131 \| L89.132 \| L89.133 \| L89.134 \| L89.136 \| L89.139 \| L89.140 \| L89.141 \| L89.142 \| L89.143 \| L89.144 \| L89.146 \| L89.149 \| L89.150 \| L89.151 \| L89.152 \| L89.153 \| L89.154 \| L89.156 \| L89.159 \| L89.200 \| L89.201 \| L89.202 \| L89.203 \| L89.204 \| L89.206 \| L89.209 \| L89.210 \| L89.211 \| L89.212 \| L89.213 \| L89.214 \| L89.216 \| L89.219 \| L89.220 \| L89.221 \| L89.222 \| L89.223 \| L89.224 \| L89.226 \| L89.229 \| L89.300 \| L89.301 \| L89.302 \| L89.303 \| L89.304 \| L89.306 \| L89.309 \| L89.310 \| L89.311 \| L89.312 \| L89.313 \| L89.314 \| L89.316 \| L89.319 \| L89.320 \| L89.321 \| L89.322 \| L89.323 \| L89.324 \| L89.326 \| L89.329 \| L89.40 \| L89.41 \| L89.42 \| L89.43 \| L89.44 \| L89.45 \| L89.46 \| L89.500 \| L89.501 \| L89.502 \| L89.503 \| L89.504 \| L89.506 \| L89.509 \| L89.510 \| L89.511 \| L89.512 \| L89.513 \| L89.514 \| L89.516 \| L89.519 \| L89.520 \| L89.521 \| L89.522 \| L89.523 \| L89.524 \| L89.526 \| L89.529 \| L89.600 \| L89.601 \| L89.602 \| L89.603 \| L89.604 \| L89.606 \| L89.609 \| L89.610 \| L89.611 \| L89.612 \| L89.613 \| L89.614 \| L89.616 \| L89.619 \| L89.620 \| L89.621 \| L89.622 \| L89.623 \| L89.624 \| L89.626 \| L89.629 \| L89.810 \| L89.811 \| L89.812 \| L89.813 \| L89.814 \| L89.816 \| L89.819 \| L89.890 \| L89.891 \| L89.892 \| L89.893 \| L89.894 \| L89.896 \| L89.899 \| L89.90 \| L89.91 \| L89.92 \| L89.93 \| L89.94 \| L89.95 \| L89.96 \| L97.101 \| L97.102 \| L97.103 \| L97.104 \| L97.105 \| L97.106 \| L97.108 \| L97.109 \| L97.111 \| L97.112 \| L97.113 \| L97.114 \| L97.115 \| L97.116 \| L97.118 \| L97.119 \| L97.121 \| L97.122 \| L97.123 \| L97.124 \| L97.125 \| L97.126 \| L97.128 \| L97.129 \| L97.201 \| L97.202 \| L97.203 \| L97.204 \| L97.205 \| L97.206 \| L97.208 \| L97.209 \| L97.211 \| L97.212 \| L97.213 \| L97.214 \| L97.215 \| L97.216 \| L97.218 \| L97.219 \| L97.221 \| L97.222 \| L97.223 \| L97.224 \| L97.225 \| L97.226 \| L97.228 \| L97.229 \| L97.301 \| L97.302 \| L97.303 \| L97.304 \| L97.305 \| L97.306 \| L97.308 \| L97.309 \| L97.311 \| L97.312 \| L97.313 \| L97.314 \| L97.315 \| L97.316 \| L97.318 \| L97.319 \| L97.321 \| L97.322 \| L97.323 \| L97.324 \| L97.325 \| L97.326 \| L97.328 \| L97.329 \| L97.401 \| L97.402 \| L97.403 \| L97.404 \| L97.405 \| L97.406 \| L97.408 \| L97.409 \| L97.411 \| L97.412 \| L97.413 \| L97.414 \| L97.415 \| L97.416 \| L97.418 \| L97.419 \| L97.421 \| L97.422 \| L97.423 \| L97.424 \| L97.425 \| L97.426 \| L97.428 \| L97.429 \| L97.501 \| L97.502 \| L97.503 \| L97.504 \| L97.505 \| L97.506 \| L97.508 \| L97.509 \| L97.511 \| L97.512 \| L97.513 \| L97.514 \| L97.515 \| L97.516 \| L97.518 \| L97.519 \| L97.521 \| L97.522 \| L97.523 \| L97.524 \| L97.525 \| L97.526 \| L97.528 \| L97.529 \| L97.801 \| L97.802 \| L97.803 \| L97.804 \| L97.805 \| L97.806 \| L97.808 \| L97.809 \| L97.811 \| L97.812 \| L97.813 \| L97.814 \| L97.815 \| L97.816 \| L97.818 \| L97.819 \| L97.821 \| L97.822 \| L97.823 \| L97.824 \| L97.825 \| L97.826 \| L97.828 \| L97.829 \| L97.901 \| L97.902 \| L97.903 \| L97.904 \| L97.905 \| L97.906 \| L97.908 \| L97.909 \| L97.911 \| L97.912 \| L97.913 \| L97.914 \| L97.915 \| L97.916 \| L97.918 \| L97.919 \| L97.921 \| L97.922 \| L97.923 \| L97.924 \| L97.925 \| L97.926 \| L97.928 \| L97.929 \| L98.411 \| L98.412 \| L98.413 \| L98.414 \| L98.415 \| L98.416 \| L98.418 \| L98.419 \| L98.421 \| L98.422 \| L98.423 \| L98.424 \| L98.425 \| L98.426 \| L98.428 \| L98.429 \| L98.491 \| L98.492 \| L98.493 \| L98.494 \| L98.495 \| L98.496 \| L98.498 \| L98.499 |
| Rheumatoid arthritis/osteoarthritis | L40.50 \| L40.51 \| L40.54 \| L40.59 \| M05.00 \| M05.011 \| M05.012 \| M05.019 \| M05.021 \| M05.022 \| M05.029 \| M05.031 \| M05.032 \| M05.039 \| M05.041 \| M05.042 \| M05.049 \| M05.051 \| M05.052 \| M05.059 \| M05.061 \| M05.062 \| M05.069 \| M05.071 \| M05.072 \| M05.079 \| M05.09 \| M05.10 \| M05.111 \| M05.112 \| M05.119 \| M05.121 \| M05.122 \| M05.129 \| M05.131 \| M05.132 \| M05.139 \| M05.141 \| M05.142 \| M05.149 \| M05.151 \| M05.152 \| M05.159 \| M05.161 \| M05.162 \| M05.169 \| M05.171 \| M05.172 \| M05.179 \| M05.19 \| M05.20 \| M05.211 \| M05.212 \| M05.219 \| M05.221 \| M05.222 \| M05.229 \| M05.231 \| M05.232 \| M05.239 \| M05.241 \| M05.242 \| M05.249 \| M05.251 \| M05.252 \| M05.259 \| M05.261 \| M05.262 \| M05.269 \| M05.271 \| M05.272 \| M05.279 \| M05.29 \| M05.30 \| M05.311 \| M05.312 \| M05.319 \| M05.321 \| M05.322 \| M05.329 \| M05.331 \| M05.332 \| M05.339 \| M05.341 \| M05.342 \| M05.349 \| M05.351 \| M05.352 \| M05.359 \| M05.361 \| M05.362 \| M05.369 \| M05.371 \| M05.372 \| M05.379 \| M05.39 \| M05.40 \| M05.411 \| M05.412 \| M05.419 \| M05.421 \| M05.422 \| M05.429 \| M05.431 \| M05.432 \| M05.439 \| M05.441 \| M05.442 \| M05.449 \| M05.451 \| M05.452 \| M05.459 \| M05.461 \| M05.462 \| M05.469 \| M05.471 \| M05.472 \| M05.479 \| M05.49 \| M05.50 \| M05.511 \| M05.512 \| M05.519 \| M05.521 \| M05.522 \| M05.529 \| M05.531 \| M05.532 \| M05.539 \| M05.541 \| M05.542 \| M05.549 \| M05.551 \| M05.552 \| M05.559 \| M05.561 \| M05.562 \| M05.569 \| M05.571 \| M05.572 \| M05.579 \| M05.59 \| M05.60 \| M05.611 \| M05.612 \| M05.619 \| M05.621 \| M05.622 \| M05.629 \| M05.631 \| M05.632 \| M05.639 \| M05.641 \| M05.642 \| M05.649 \| M05.651 \| M05.652 \| M05.659 \| M05.661 \| M05.662 \| M05.669 \| M05.671 \| M05.672 \| M05.679 \| M05.69 \| M05.70 \| M05.711 \| M05.712 \| M05.719 \| M05.721 \| M05.722 \| M05.729 \| M05.731 \| M05.732 \| M05.739 \| M05.741 \| M05.742 \| M05.749 \| M05.751 \| M05.752 \| M05.759 \| M05.761 \| M05.762 \| M05.769 \| M05.771 \| M05.772 \| M05.779 \| M05.79 \| M05.7A \| M05.80 \| M05.811 \| M05.812 \| M05.819 \| M05.821 \| M05.822 \| M05.829 \| M05.831 \| M05.832 \| M05.839 \| M05.841 \| M05.842 \| M05.849 \| M05.851 \| M05.852 \| M05.859 \| M05.861 \| M05.862 \| M05.869 \| M05.871 \| M05.872 \| M05.879 \| M05.89 \| M05.8A \| M05.9 \| M06.00 \| M06.011 \| M06.012 \| M06.019 \| M06.021 \| M06.022 \| M06.029 \| M06.031 \| M06.032 \| M06.039 \| M06.041 \| M06.042 \| M06.049 \| M06.051 \| M06.052 \| M06.059 \| M06.061 \| M06.062 \| M06.069 \| M06.071 \| M06.072 \| M06.079 \| M06.08 \| M06.09 \| M06.0A \| M06.1 \| M06.20 \| M06.211 \| M06.212 \| M06.219 \| M06.221 \| M06.222 \| M06.229 \| M06.231 \| M06.232 \| M06.239 \| M06.241 \| M06.242 \| M06.249 \| M06.251 \| M06.252 \| M06.259 \| M06.261 \| M06.262 \| M06.269 \| M06.271 \| M06.272 \| M06.279 \| M06.28 \| M06.29 \| M06.30 \| M06.311 \| M06.312 \| M06.319 \| M06.321 \| M06.322 \| M06.329 \| M06.331 \| M06.332 \| M06.339 \| M06.341 \| M06.342 \| M06.349 \| M06.351 \| M06.352 \| M06.359 \| M06.361 \| M06.362 \| M06.369 \| M06.371 \| M06.372 \| M06.379 \| M06.38 \| M06.39 \| M06.80 \| M06.811 \| M06.812 \| M06.819 \| M06.821 \| M06.822 \| M06.829 \| M06.831 \| M06.832 \| M06.839 \| M06.841 \| M06.842 \| M06.849 \| M06.851 \| M06.852 \| M06.859 \| M06.861 \| M06.862 \| M06.869 \| M06.871 \| M06.872 \| M06.879 \| M06.88 \| M06.89 \| M06.8A \| M06.9 \| M08.00 \| M08.011 \| M08.012 \| M08.019 \| M08.021 \| M08.022 \| M08.029 \| M08.031 \| M08.032 \| M08.039 \| M08.041 \| M08.042 \| M08.049 \| M08.051 \| M08.052 \| M08.059 \| M08.061 \| M08.062 \| M08.069 \| M08.071 \| M08.072 \| M08.079 \| M08.08 \| M08.09 \| M08.0A \| M08.1 \| M08.20 \| M08.211 \| M08.212 \| M08.219 \| M08.221 \| M08.222 \| M08.229 \| M08.231 \| M08.232 \| M08.239 \| M08.241 \| M08.242 \| M08.249 \| M08.251 \| M08.252 \| M08.259 \| M08.261 \| M08.262 \| M08.269 \| M08.271 \| M08.272 \| M08.279 \| M08.28 \| M08.29 \| M08.2A \| M08.3 \| M08.40 \| M08.411 \| M08.412 \| M08.419 \| M08.421 \| M08.422 \| M08.429 \| M08.431 \| M08.432 \| M08.439 \| M08.441 \| M08.442 \| M08.449 \| M08.451 \| M08.452 \| M08.459 \| M08.461 \| M08.462 \| M08.469 \| M08.471 \| M08.472 \| M08.479 \| M08.48 \| M08.4A \| M08.80 \| M08.811 \| M08.812 \| M08.819 \| M08.821 \| M08.822 \| M08.829 \| M08.831 \| M08.832 \| M08.839 \| M08.841 \| M08.842 \| M08.849 \| M08.851 \| M08.852 \| M08.859 \| M08.861 \| M08.862 \| M08.869 \| M08.871 \| M08.872 \| M08.879 \| M08.88 \| M08.89 \| M08.90 \| M08.911 \| M08.912 \| M08.919 \| M08.921 \| M08.922 \| M08.929 \| M08.931 \| M08.932 \| M08.939 \| M08.941 \| M08.942 \| M08.949 \| M08.951 \| M08.952 \| M08.959 \| M08.961 \| M08.962 \| M08.969 \| M08.971 \| M08.972 \| M08.979 \| M08.98 \| M08.99 \| M08.9A \| M15.0 \| M15.1 \| M15.2 \| M15.3 \| M15.4 \| M15.8 \| M15.9 \| M16.0 \| M16.10 \| M16.11 \| M16.12 \| M16.2 \| M16.30 \| M16.31 \| M16.32 \| M16.4 \| M16.50 \| M16.51 \| M16.52 \| M16.6 \| M16.7 \| M16.9 \| M17.0 \| M17.10 \| M17.11 \| M17.12 \| M17.2 \| M17.30 \| M17.31 \| M17.32 \| M17.4 \| M17.5 \| M17.9 \| M18.0 \| M18.10 \| M18.11 \| M18.12 \| M18.2 \| M18.30 \| M18.31 \| M18.32 \| M18.4 \| M18.50 \| M18.51 \| M18.52 \| M18.9 \| M19.011 \| M19.012 \| M19.019 \| M19.021 \| M19.022 \| M19.029 \| M19.031 \| M19.032 \| M19.039 \| M19.041 \| M19.042 \| M19.049 \| M19.071 \| M19.072 \| M19.079 \| M19.09 \| M19.111 \| M19.112 \| M19.119 \| M19.121 \| M19.122 \| M19.129 \| M19.131 \| M19.132 \| M19.139 \| M19.141 \| M19.142 \| M19.149 \| M19.171 \| M19.172 \| M19.179 \| M19.19 \| M19.211 \| M19.212 \| M19.219 \| M19.221 \| M19.222 \| M19.229 \| M19.231 \| M19.232 \| M19.239 \| M19.241 \| M19.242 \| M19.249 \| M19.271 \| M19.272 \| M19.279 \| M19.29 \| M19.90 \| M19.91 \| M19.92 \| M19.93 \| M45.0 \| M45.1 \| M45.2 \| M45.3 \| M45.4 \| M45.5 \| M45.6 \| M45.7 \| M45.8 \| M45.9 \| M45.A0 \| M45.A1 \| M45.A2 \| M45.A3 \| M45.A4 \| M45.A5 \| M45.A6 \| M45.A7 \| M45.A8 \| M45.AB \| M46.80 \| M46.81 \| M46.82 \| M46.83 \| M46.84 \| M46.85 \| M46.86 \| M46.87 \| M46.88 \| M46.89 \| M46.90 \| M46.91 \| M46.92 \| M46.93 \| M46.94 \| M46.95 \| M46.96 \| M46.97 \| M46.98 \| M46.99 \| M47.011 \| M47.012 \| M47.013 \| M47.014 \| M47.015 \| M47.016 \| M47.019 \| M47.021 \| M47.022 \| M47.029 \| M47.10 \| M47.11 \| M47.12 \| M47.13 \| M47.14 \| M47.15 \| M47.16 \| M47.20 \| M47.21 \| M47.22 \| M47.23 \| M47.24 \| M47.25 \| M47.26 \| M47.27 \| M47.28 \| M47.811 \| M47.812 \| M47.813 \| M47.814 \| M47.815 \| M47.816 \| M47.817 \| M47.818 \| M47.819 \| M47.891 \| M47.892 \| M47.893 \| M47.894 \| M47.895 \| M47.896 \| M47.897 \| M47.898 \| M47.899 \| M47.9 \| M48.8X1 \| M48.8X2 \| M48.8X3 \| M48.8X4 \| M48.8X5 \| M48.8X6 \| M48.8X7 \| M48.8X8 \| M48.8X9 |
| Schizophrenia and other psychotic disorders | F06.0 \| F06.2 \| F20.0 \| F20.1 \| F20.2 \| F20.3 \| F20.5 \| F20.81 \| F20.89 \| F20.9 \| F22 \| F23 \| F24 \| F25.0 \| F25.1 \| F25.8 \| F25.9 \| F28 \| F29 \| F32.3 \| F44.89 |
| Spina bifida and other congenital anomalies of the nervous system | G90.1 \| Q00.0 \| Q00.1 \| Q00.2 \| Q01.0 \| Q01.1 \| Q01.2 \| Q01.8 \| Q01.9 \| Q02 \| Q03.0 \| Q03.1 \| Q03.8 \| Q03.9 \| Q04.0 \| Q04.1 \| Q04.2 \| Q04.3 \| Q04.4 \| Q04.5 \| Q04.6 \| Q04.8 \| Q04.9 \| Q05.0 \| Q05.1 \| Q05.2 \| Q05.3 \| Q05.4 \| Q05.5 \| Q05.6 \| Q05.7 \| Q05.8 \| Q05.9 \| Q06.0 \| Q06.1 \| Q06.2 \| Q06.3 \| Q06.4 \| Q06.8 \| Q06.9 \| Q07.00 \| Q07.01 \| Q07.02 \| Q07.03 \| Q07.8 \| Q07.9 |
| Spinal cord injury | G96.11 \| S12.000A \| S12.000B \| S12.001A \| S12.001B \| S12.100A \| S12.100B \| S12.101A \| S12.101B \| S12.200A \| S12.200B \| S12.201A \| S12.201B \| S12.300A \| S12.300B \| S12.301A \| S12.301B \| S12.400A \| S12.400B \| S12.401A \| S12.401B \| S12.500A \| S12.500B \| S12.501A \| S12.501B \| S12.600A \| S12.600B \| S12.601A \| S12.601B \| S12.9XXA \| S13.113A \| S14.0XXA \| S14.0XXS \| S14.101A \| S14.101S \| S14.102A \| S14.102S \| S14.103A \| S14.103S \| S14.104A \| S14.104S \| S14.105A \| S14.105S \| S14.106A \| S14.106S \| S14.107A \| S14.107S \| S14.108A \| S14.108S \| S14.109A \| S14.109S \| S14.111A \| S14.111S \| S14.112A \| S14.112S \| S14.113S \| S14.114A \| S14.114S \| S14.115A \| S14.115S \| S14.116A \| S14.116S \| S14.117A \| S14.117S \| S14.118A \| S14.118S \| S14.119A \| S14.119S \| S14.121A \| S14.121S \| S14.122A \| S14.122S \| S14.123A \| S14.123S \| S14.124A \| S14.124S \| S14.125A \| S14.125S \| S14.126A \| S14.126S \| S14.127A \| S14.127S \| S14.128A \| S14.128S \| S14.129A \| S14.129S \| S14.131A \| S14.131S \| S14.132A \| S14.132S \| S14.133A \| S14.133S \| S14.134A \| S14.134S \| S14.135A \| S14.135S \| S14.136A \| S14.136S \| S14.137A \| S14.137S \| S14.138A \| S14.138S \| S14.139A \| S14.139S \| S14.141A \| S14.141S \| S14.142A \| S14.142S \| S14.143A \| S14.143S \| S14.144A \| S14.144S \| S14.145A \| S14.145S \| S14.146A \| S14.146S \| S14.147A \| S14.147S \| S14.148A \| S14.148S \| S14.149A \| S14.149S \| S14.151A \| S14.151S \| S14.152A \| S14.152S \| S14.153A \| S14.153S \| S14.154A \| S14.154S \| S14.155A \| S14.155S \| S14.156A \| S14.156S \| S14.157A \| S14.157S \| S14.158A \| S14.158S \| S14.159A \| S14.159S \| S22.009A \| S22.009B \| S22.019A \| S22.019B \| S22.029A \| S22.029B \| S22.039A \| S22.039B \| S22.049A \| S22.049B \| S22.059A \| S22.059B \| S22.069A \| S22.069B \| S22.079A \| S22.079B \| S22.089A \| S22.089B \| S24.0XXA \| S24.0XXS \| S24.101A \| S24.101S \| S24.102A \| S24.102S \| S24.103A \| S24.103S \| S24.104A \| S24.104S \| S24.109A \| S24.109S \| S24.111A \| S24.111S \| S24.112A \| S24.112S \| S24.113A \| S24.113S \| S24.114A \| S24.114S \| S24.119A \| S24.119S \| S24.131A \| S24.131S \| S24.132A \| S24.132S \| S24.133A \| S24.133S \| S24.134A \| S24.134S \| S24.139A \| S24.139S \| S24.141A \| S24.141S \| S24.142A \| S24.142S \| S24.143A \| S24.143S \| S24.144A \| S24.144S \| S24.149A \| S24.149S \| S24.151A \| S24.151S \| S24.152A \| S24.152S \| S24.153A \| S24.153S \| S24.154A \| S24.154S \| S24.159A \| S24.159S \| S32.009A \| S32.009B \| S32.019A \| S32.019B \| S32.029A \| S32.029B \| S32.039A \| S32.039B \| S32.049A \| S32.049B \| S32.059A \| S32.059B \| S32.10XA \| S32.10XB \| S32.2XXA \| S32.2XXB \| S34.01XA \| S34.01XS \| S34.02XA \| S34.02XS \| S34.101A \| S34.101S \| S34.102A \| S34.102S \| S34.103A \| S34.103S \| S34.104A \| S34.104S \| S34.105A \| S34.105S \| S34.109A \| S34.109S \| S34.111A \| S34.111S \| S34.112A \| S34.112S \| S34.113A \| S34.113S \| S34.114A \| S34.114S \| S34.115A \| S34.115S \| S34.119A \| S34.119S \| S34.121A \| S34.121S \| S34.122A \| S34.122S \| S34.123A \| S34.123S \| S34.124A \| S34.124S \| S34.125A \| S34.125S \| S34.129A \| S34.129S \| S34.131A \| S34.131S \| S34.132A \| S34.132S \| S34.139A \| S34.139S \| S34.3XXA |
| Stroke/transient ischemic attack | G45.0 \| G45.1 \| G45.2 \| G45.3 \| G45.8 \| G45.9 \| G46.0 \| G46.1 \| G46.2 \| G46.3 \| G46.4 \| G46.5 \| G46.6 \| G46.7 \| G46.8 \| G97.31 \| G97.32 \| I60.00 \| I60.01 \| I60.02 \| I60.10 \| I60.11 \| I60.12 \| I60.2 \| I60.20 \| I60.21 \| I60.22 \| I60.30 \| I60.31 \| I60.32 \| I60.4 \| I60.50 \| I60.51 \| I60.52 \| I60.6 \| I60.7 \| I60.8 \| I60.9 \| I61.0 \| I61.1 \| I61.2 \| I61.3 \| I61.4 \| I61.5 \| I61.6 \| I61.8 \| I61.9 \| I62.00 \| I62.01 \| I62.02 \| I62.9 \| I63.00 \| I63.011 \| I63.012 \| I63.013 \| I63.019 \| I63.02 \| I63.031 \| I63.032 \| I63.033 \| I63.039 \| I63.09 \| I63.10 \| I63.111 \| I63.112 \| I63.113 \| I63.119 \| I63.12 \| I63.131 \| I63.132 \| I63.133 \| I63.139 \| I63.19 \| I63.20 \| I63.211 \| I63.212 \| I63.213 \| I63.219 \| I63.22 \| I63.231 \| I63.232 \| I63.233 \| I63.239 \| I63.29 \| I63.30 \| I63.311 \| I63.312 \| I63.313 \| I63.319 \| I63.321 \| I63.322 \| I63.323 \| I63.329 \| I63.331 \| I63.332 \| I63.333 \| I63.339 \| I63.341 \| I63.342 \| I63.343 \| I63.349 \| I63.39 \| I63.40 \| I63.411 \| I63.412 \| I63.413 \| I63.419 \| I63.421 \| I63.422 \| I63.423 \| I63.429 \| I63.431 \| I63.432 \| I63.433 \| I63.439 \| I63.441 \| I63.442 \| I63.443 \| I63.449 \| I63.49 \| I63.50 \| I63.511 \| I63.512 \| I63.513 \| I63.519 \| I63.521 \| I63.522 \| I63.523 \| I63.529 \| I63.531 \| I63.532 \| I63.533 \| I63.539 \| I63.541 \| I63.542 \| I63.543 \| I63.549 \| I63.59 \| I63.6 \| I63.8 \| I63.81 \| I63.89 \| I63.9 \| I67.841 \| I67.848 \| I67.89 \| I97.810 \| I97.811 \| I97.820 \| I97.821 |
| Substance use disorders^4^ | F10.10 \| F10.120 \| F10.121 \| F10.129 \| F10.130 \| F10.131 \| F10.132 \| F10.139 \| F10.14 \| F10.150 \| F10.151 \| F10.159 \| F10.180 \| F10.181 \| F10.182 \| F10.188 \| F10.19 \| F10.20 \| F10.220 \| F10.221 \| F10.229 \| F10.230 \| F10.231 \| F10.232 \| F10.239 \| F10.24 \| F10.250 \| F10.251 \| F10.259 \| F10.26 \| F10.27 \| F10.280 \| F10.281 \| F10.282 \| F10.288 \| F10.29 \| F10.920 \| F10.921 \| F10.929 \| F10.930 \| F10.931 \| F10.932 \| F10.939 \| F10.94 \| F10.950 \| F10.951 \| F10.959 \| F10.96 \| F10.97 \| F10.980 \| F10.981 \| F10.982 \| F10.988 \| F10.99 \| F11.10 \| F11.120 \| F11.121 \| F11.122 \| F11.129 \| F11.13 \| F11.14 \| F11.150 \| F11.151 \| F11.159 \| F11.181 \| F11.182 \| F11.188 \| F11.19 \| F11.20 \| F11.220 \| F11.221 \| F11.222 \| F11.229 \| F11.23 \| F11.24 \| F11.250 \| F11.251 \| F11.259 \| F11.281 \| F11.282 \| F11.288 \| F11.29 \| F11.90 \| F11.920 \| F11.921 \| F11.922 \| F11.929 \| F11.93 \| F11.94 \| F11.950 \| F11.951 \| F11.959 \| F11.981 \| F11.982 \| F11.988 \| F11.99 \| F12.10 \| F12.120 \| F12.121 \| F12.122 \| F12.129 \| F12.13 \| F12.150 \| F12.151 \| F12.159 \| F12.180 \| F12.188 \| F12.19 \| F12.20 \| F12.220 \| F12.221 \| F12.222 \| F12.229 \| F12.250 \| F12.251 \| F12.259 \| F12.280 \| F12.288 \| F12.29 \| F12.90 \| F12.920 \| F12.921 \| F12.922 \| F12.929 \| F12.950 \| F12.951 \| F12.959 \| F12.980 \| F12.988 \| F12.99 \| F13.10 \| F13.120 \| F13.121 \| F13.129 \| F13.130 \| F13.131 \| F13.132 \| F13.139 \| F13.14 \| F13.150 \| F13.151 \| F13.159 \| F13.180 \| F13.181 \| F13.182 \| F13.188 \| F13.19 \| F13.20 \| F13.220 \| F13.221 \| F13.229 \| F13.230 \| F13.231 \| F13.232 \| F13.239 \| F13.24 \| F13.250 \| F13.251 \| F13.259 \| F13.26 \| F13.27 \| F13.280 \| F13.281 \| F13.282 \| F13.288 \| F13.29 \| F13.90 \| F13.920 \| F13.921 \| F13.929 \| F13.930 \| F13.931 \| F13.932 \| F13.939 \| F13.94 \| F13.950 \| F13.951 \| F13.959 \| F13.96 \| F13.97 \| F13.980 \| F13.981 \| F13.982 \| F13.988 \| F13.99 \| F14.10 \| F14.120 \| F14.121 \| F14.122 \| F14.129 \| F14.13 \| F14.14 \| F14.150 \| F14.151 \| F14.159 \| F14.180 \| F14.181 \| F14.182 \| F14.188 \| F14.19 \| F14.20 \| F14.220 \| F14.221 \| F14.222 \| F14.229 \| F14.23 \| F14.24 \| F14.250 \| F14.251 \| F14.259 \| F14.280 \| F14.281 \| F14.282 \| F14.288 \| F14.29 \| F14.90 \| F14.920 \| F14.921 \| F14.922 \| F14.929 \| F14.93 \| F14.94 \| F14.950 \| F14.951 \| F14.959 \| F14.980 \| F14.981 \| F14.982 \| F14.988 \| F14.99 \| F15.10 \| F15.120 \| F15.121 \| F15.122 \| F15.129 \| F15.13 \| F15.14 \| F15.150 \| F15.151 \| F15.159 \| F15.180 \| F15.181 \| F15.182 \| F15.188 \| F15.19 \| F15.20 \| F15.220 \| F15.221 \| F15.222 \| F15.229 \| F15.23 \| F15.24 \| F15.250 \| F15.251 \| F15.259 \| F15.280 \| F15.281 \| F15.282 \| F15.288 \| F15.29 \| F15.90 \| F15.920 \| F15.921 \| F15.922 \| F15.929 \| F15.93 \| F15.94 \| F15.950 \| F15.951 \| F15.959 \| F15.980 \| F15.981 \| F15.982 \| F15.988 \| F15.99 \| F16.10 \| F16.120 \| F16.121 \| F16.122 \| F16.129 \| F16.14 \| F16.150 \| F16.151 \| F16.159 \| F16.180 \| F16.183 \| F16.188 \| F16.19 \| F16.20 \| F16.220 \| F16.221 \| F16.229 \| F16.24 \| F16.250 \| F16.251 \| F16.259 \| F16.280 \| F16.283 \| F16.288 \| F16.29 \| F16.90 \| F16.920 \| F16.921 \| F16.929 \| F16.94 \| F16.950 \| F16.951 \| F16.959 \| F16.980 \| F16.983 \| F16.988 \| F16.99 \| F17.200 \| F17.201 \| F17.203 \| F17.208 \| F17.209 \| F17.210 \| F17.211 \| F17.213 \| F17.218 \| F17.219 \| F17.220 \| F17.221 \| F17.223 \| F17.228 \| F17.229 \| F17.290 \| F17.291 \| F17.293 \| F17.298 \| F17.299 \| F18.10 \| F18.120 \| F18.121 \| F18.129 \| F18.14 \| F18.150 \| F18.151 \| F18.159 \| F18.17 \| F18.180 \| F18.188 \| F18.19 \| F18.20 \| F18.220 \| F18.221 \| F18.229 \| F18.24 \| F18.250 \| F18.251 \| F18.259 \| F18.27 \| F18.280 \| F18.288 \| F18.29 \| F18.90 \| F18.920 \| F18.921 \| F18.929 \| F18.94 \| F18.950 \| F18.951 \| F18.959 \| F18.97 \| F18.980 \| F18.988 \| F18.99 \| F19.10 \| F19.120 \| F19.121 \| F19.122 \| F19.129 \| F19.130 \| F19.131 \| F19.132 \| F19.139 \| F19.14 \| F19.150 \| F19.151 \| F19.159 \| F19.16 \| F19.17 \| F19.180 \| F19.181 \| F19.182 \| F19.188 \| F19.19 \| F19.20 \| F19.220 \| F19.221 \| F19.222 \| F19.229 \| F19.230 \| F19.231 \| F19.232 \| F19.239 \| F19.24 \| F19.250 \| F19.251 \| F19.259 \| F19.26 \| F19.27 \| F19.280 \| F19.281 \| F19.282 \| F19.288 \| F19.29 \| F19.90 \| F19.920 \| F19.921 \| F19.922 \| F19.929 \| F19.930 \| F19.931 \| F19.932 \| F19.939 \| F19.94 \| F19.950 \| F19.951 \| F19.959 \| F19.96 \| F19.97 \| F19.980 \| F19.981 \| F19.982 \| F19.988 \| F19.99 \| F55.0 \| F55.1 \| F55.2 \| F55.3 \| F55.4 \| F55.8 \| G62.1 \| K29.20 \| K29.21 \| O35.5XX0 \| O35.5XX1 \| O35.5XX2 \| O35.5XX3 \| O35.5XX4 \| O35.5XX5 \| O35.5XX9 \| O99.320 \| O99.321 \| O99.322 \| O99.323 \| O99.324 \| O99.325 \| O99.330 \| O99.331 \| O99.332 \| O99.333 \| O99.334 \| O99.335 \| T40.0X1A \| T40.0X1D \| T40.0X1S \| T40.0X2A \| T40.0X2D \| T40.0X2S \| T40.0X3A \| T40.0X3D \| T40.0X3S \| T40.0X4A \| T40.0X4D \| T40.0X4S \| T40.0X5A \| T40.0X5D \| T40.0X5S \| T40.1X1A \| T40.1X1D \| T40.1X1S \| T40.1X2A \| T40.1X2D \| T40.1X2S \| T40.1X3A \| T40.1X3D \| T40.1X3S \| T40.1X4A \| T40.1X4D \| T40.1X4S \| T40.2X1A \| T40.2X1D \| T40.2X1S \| T40.2X2A \| T40.2X2D \| T40.2X2S \| T40.2X3A \| T40.2X3D \| T40.2X3S \| T40.2X4A \| T40.2X4D \| T40.2X4S \| T40.2X5A \| T40.2X5D \| T40.2X5S \| T40.3X1A \| T40.3X1D \| T40.3X1S \| T40.3X2A \| T40.3X2D \| T40.3X2S \| T40.3X3A \| T40.3X3D \| T40.3X3S \| T40.3X4A \| T40.3X4D \| T40.3X4S \| T40.3X5A \| T40.3X5D \| T40.3X5S \| T40.411A \| T40.411D \| T40.411S \| T40.412A \| T40.412D \| T40.412S \| T40.413A \| T40.413D \| T40.413S \| T40.414A \| T40.414D \| T40.414S \| T40.415A \| T40.415D \| T40.415S \| T40.421A \| T40.421D \| T40.421S \| T40.422A \| T40.422D \| T40.422S \| T40.423A \| T40.423D \| T40.423S \| T40.424A \| T40.424D \| T40.424S \| T40.425A \| T40.425D \| T40.425S \| T40.491A \| T40.491D \| T40.491S \| T40.492A \| T40.492D \| T40.492S \| T40.493A \| T40.493D \| T40.493S \| T40.494A \| T40.494D \| T40.494S \| T40.495A \| T40.495D \| T40.495S \| T40.4X1A \| T40.4X1D \| T40.4X1S \| T40.4X2A \| T40.4X2D \| T40.4X2S \| T40.4X3A \| T40.4X3D \| T40.4X3S \| T40.4X4A \| T40.4X4D \| T40.4X4S \| T40.4X5A \| T40.4X5D \| T40.4X5S \| T40.601A \| T40.601D \| T40.601S \| T40.602A \| T40.602D \| T40.602S \| T40.603A \| T40.603D \| T40.603S \| T40.604A \| T40.604D \| T40.604S \| T40.605A \| T40.605D \| T40.605S \| T40.691A \| T40.691D \| T40.691S \| T40.692A \| T40.692D \| T40.692S \| T40.693A \| T40.693D \| T40.693S \| T40.694A \| T40.694D \| T40.694S \| T40.695A \| T40.695D \| T40.695S \| T40.711A \| T40.721A \| T40.7X1A \| T40.8X1A \| T40.901A \| T40.991A \| T51.0X1A \| T51.0X2A \| T51.0X3A \| T51.0X4A \| T65.211A \| T65.212A \| T65.213A \| T65.214A \| T65.221A \| T65.222A \| T65.223A \| T65.224A \| T65.291A \| T65.292A \| T65.293A \| T65.294A \| Z72.0 |
| Traumatic brain injury and nonpsychotic mental disorders due to brain damage | F07.0 \| F07.81 \| F07.89 \| F48.2 \| S04.011S \| S04.012S \| S04.019S \| S04.02XS \| S04.031S \| S04.032S \| S04.039S \| S04.041S \| S04.042S \| S04.049S \| S04.10XS \| S04.11XS \| S04.12XS \| S04.20XS \| S04.21XS \| S04.22XS \| S04.30XS \| S04.31XS \| S04.32XS \| S04.40XS \| S04.41XS \| S04.42XS \| S04.50XS \| S04.51XS \| S04.52XS \| S04.60XS \| S04.61XS \| S04.62XS \| S04.70XS \| S04.71XS \| S04.72XS \| S04.811S \| S04.812S \| S04.819S \| S04.891S \| S04.892S \| S04.899S \| S04.9XXS \| S06.0X0S \| S06.0X1S \| S06.0X2S \| S06.0X3S \| S06.0X4S \| S06.0X5S \| S06.0X6S \| S06.0X7S \| S06.0X8S \| S06.0X9S \| S06.0XAS \| S06.1X0S \| S06.1X1S \| S06.1X2S \| S06.1X3S \| S06.1X4S \| S06.1X5S \| S06.1X6S \| S06.1X7S \| S06.1X8S \| S06.1X9S \| S06.1XAS \| S06.2X0S \| S06.2X1S \| S06.2X2S \| S06.2X3S \| S06.2X4S \| S06.2X5S \| S06.2X6S \| S06.2X7S \| S06.2X8S \| S06.2X9S \| S06.2XAS \| S06.300S \| S06.301S \| S06.302S \| S06.303S \| S06.304S \| S06.305S \| S06.306S \| S06.307S \| S06.308S \| S06.309S \| S06.30AS \| S06.310S \| S06.311S \| S06.312S \| S06.313S \| S06.314S \| S06.315S \| S06.316S \| S06.317S \| S06.318S \| S06.319S \| S06.31AS \| S06.320S \| S06.321S \| S06.322S \| S06.323S \| S06.324S \| S06.325S \| S06.326S \| S06.327S \| S06.328S \| S06.329S \| S06.32AS \| S06.330S \| S06.331S \| S06.332S \| S06.333S \| S06.334S \| S06.335S \| S06.336S \| S06.337S \| S06.338S \| S06.339S \| S06.33AS \| S06.340S \| S06.341S \| S06.342S \| S06.343S \| S06.344S \| S06.345S \| S06.346S \| S06.347S \| S06.348S \| S06.349S \| S06.34AS \| S06.350S \| S06.351S \| S06.352S \| S06.353S \| S06.354S \| S06.355S \| S06.356S \| S06.357S \| S06.358S \| S06.359S \| S06.35AS \| S06.360S \| S06.361S \| S06.362S \| S06.363S \| S06.364S \| S06.365S \| S06.366S \| S06.367S \| S06.368S \| S06.369S \| S06.36AS \| S06.370S \| S06.371S \| S06.372S \| S06.373S \| S06.374S \| S06.375S \| S06.376S \| S06.377S \| S06.378S \| S06.379S \| S06.37AS \| S06.380S \| S06.381S \| S06.382S \| S06.383S \| S06.384S \| S06.385S \| S06.386S \| S06.387S \| S06.388S \| S06.389S \| S06.38AS \| S06.4X0S \| S06.4X1S \| S06.4X2S \| S06.4X3S \| S06.4X4S \| S06.4X5S \| S06.4X6S \| S06.4X7S \| S06.4X8S \| S06.4X9S \| S06.4XAS \| S06.5X0S \| S06.5X1S \| S06.5X2S \| S06.5X3S \| S06.5X4S \| S06.5X5S \| S06.5X6S \| S06.5X7S \| S06.5X8S \| S06.5X9S \| S06.5XAS \| S06.6X0S \| S06.6X1S \| S06.6X2S \| S06.6X3S \| S06.6X4S \| S06.6X5S \| S06.6X6S \| S06.6X7S \| S06.6X8S \| S06.6X9S \| S06.6XAS \| S06.810S \| S06.811S \| S06.812S \| S06.813S \| S06.814S \| S06.815S \| S06.816S \| S06.817S \| S06.818S \| S06.819S \| S06.81AS \| S06.820S \| S06.821S \| S06.822S \| S06.823S \| S06.824S \| S06.825S \| S06.826S \| S06.827S \| S06.828S \| S06.829S \| S06.82AS \| S06.890S \| S06.891S \| S06.892S \| S06.893S \| S06.894S \| S06.895S \| S06.896S \| S06.897S \| S06.898S \| S06.899S \| S06.89AS \| S06.8A0S \| S06.8A1S \| S06.8A2S \| S06.8A3S \| S06.8A4S \| S06.8A5S \| S06.8A6S \| S06.8A9S \| S06.8AAS \| S06.9X0S \| S06.9X1S \| S06.9X2S \| S06.9X3S \| S06.9X4S \| S06.9X5S \| S06.9X6S \| S06.9X7S \| S06.9X8S \| S06.9X9S \| S06.9XAS \| S06.A0XS \| S06.A1XS |
| Viral hepatitis | B15.0 \| B15.9 \| B16.0 \| B16.1 \| B16.2 \| B16.9 \| B17.0 \| B17.10 \| B17.11 \| B17.2 \| B17.8 \| B17.9 \| B18.0 \| B18.1 \| B18.2 \| B18.8 \| B18.9 \| B19.0 \| B19.10 \| B19.11 \| B19.20 \| B19.21 \| B19.9 \| Z22.50 \| Z22.51 \| Z22.52 \| Z22.59 |
| ADHD=Attention-deficit/hyperactivity disorder; HIV/AIDS=Human immunodeficiency virus/Acquired immunodeficiency syndrome.  International Classification of Disease-10 Revision (ICD-10) codes from the Centers for Medicare and Medicaid Services Chronic Condition Warehouse were used identify diagnosis of a chronic condition (5-7).  ^1^Includes ICD-10 codes for “anemia” and “sickle cell anemia”  ^2^Includes ICD-10 codes for “anxiety disorders” and “post-traumatic stress disorder”  ^3^Includes ICD-10 codes for “learning disabilities” and “other developmental delays”  ^4^Includes ICD-10 codes for “alcohol use disorders”; “drug use disorders”; opioid use disorder 1, 2, 3 and 4; and “tobacco use disorders”, excluding Z71.41, Z71.42, Z71.51, Z71.52, and Z71.6 | |

| **Supplementary Table 3. Most frequently diagnosed multiple chronic condition combinations in each age category** | |
| --- | --- |
| **Condition combinations** | **n** |
| **Early adulthood, 18-39 years** (n=40,237) | |
| Anxiety disorders; Depression, bipolar, or other depressive mood disorders | 845 |
| Anemia; Obesity | 355 |
| Anxiety disorders; Depression, bipolar, or other depressive mood disorders; Substance use disorders | 340 |
| Anxiety disorders; Fibromyalgia, chronic pain and fatigue | 238 |
| Anxiety disorders; Depression, bipolar, or other depressive mood disorders; Fibromyalgia, chronic pain and fatigue | 216 |
| **Middle adulthood, 40-49 years** (n=28,906) | |
| Fibromyalgia, chronic pain and fatigue; Rheumatoid arthritis/osteoarthritis | 204 |
| Anxiety disorders; Depression, bipolar, or other depressive mood disorders | 168 |
| Hyperlipidemia; Hypertension | 104 |
| Hypertension; Substance use disorders | 103 |
| Hypertension; Obesity | 84 |
| **Late middle adulthood, 50-64 years** (n=66,705) | |
| Fibromyalgia, chronic pain and fatigue; Rheumatoid arthritis/osteoarthritis | 457 |
| Hyperlipidemia; Hypertension | 385 |
| Hypertension; Substance use disorders | 216 |
| Diabetes; Hyperlipidemia; Hypertension | 204 |
| Diabetes; Hypertension | 173 |
| **Late adulthood, 65-74 years** (n=35,549) | |
| Hyperlipidemia; Hypertension | 200 |
| Fibromyalgia, chronic pain and fatigue; Rheumatoid arthritis/osteoarthritis | 182 |
| Cataract; Glaucoma | 94 |
| Hyperlipidemia; Hypertension; Rheumatoid arthritis/osteoarthritis | 85 |
| Hypertension; Rheumatoid arthritis/osteoarthritis | 84 |
| **Advanced old age, 75-89 years** (n=11,289) | |
| Hyperlipidemia; Hypertension | 59 |
| Fibromyalgia, chronic pain and fatigue; Rheumatoid arthritis/osteoarthritis | 46 |
| Cataract; Glaucoma | 44 |
| Hyperlipidemia; Hypertension; Ischemic heart disease | 38 |
| Diabetes; Hyperlipidemia; Hypertension | 26 |
